# ﻿A review of the genus *Hesperosoma* Scheerpeltz (Coleoptera, Staphylinidae, Staphylininae) of China

**DOI:** 10.3897/zookeys.1075.75799

**Published:** 2021-12-07

**Authors:** Yu-Jie Cai1, Liang Tang1, Harald Schillhammer2

**Affiliations:** 1 College of Science, Shanghai Normal University, 100 Guilin Road, 1 Shanghai Normal University Shanghai China; 2 st Naturhistorisches Museum Wien Wien Austria; 3 Educational Building 323 Room, Shanghai, 200234, China Shanghai Normal University Shanghai China; 4 Naturhistorisches Museum Wien, Burgring 7, A – 1010 Wien, Austria Naturhistorisches Museum Wien Wien Austria

**Keywords:** Identification key, new records, new species

## Abstract

A review of 16 species of *Hesperosoma* Scheerpeltz from China is presented. Five new species are described: H.(s.str.)chenchangchini**sp. nov.** from Yunnan, H.(s.str.)languidum**sp. nov.** from Yunnan, H.(s.str.)motuoense**sp. nov.** from Xizang, H. (Paramichrotus) parvioculatum**sp. nov.** from Hubei, Hunan and H.(s.str.)xizangense**sp. nov.** from Xizang. Two species are new to China: H. (Paramichrotus) brunkei Schillhammer, 2015 from Yunnan and H.(s.str.)kleebergi Schillhammer, 2009 from Xizang. Females of H. (Paramichrotus) alexpuchneri Schillhammer, 2009, H. (Paramichrotus) guizhouense Schillhammer, 2018 and H.(s.str.)flavoterminale Schillhammer, 2004 are described for the first time. Habitus and diagnostic characters of all species are photographed and a key to Chinese species of *Hesperosoma* is provided.

## ﻿Introduction

*Hesperosoma* Scheerpeltz, 1965 is an Asian genus with 29 known species (Schillhammer 2004, 2009, 2015, 2018) in the subtribe Anisolinina Hayashi, 1993 of the tribe Staphylinini. The species of this genus may be easily distinguished from members of related genera (*Philomyceta*, *Hesperoschema*) by segment 2 of maxillary palpi less distinctly or hardly dilated, abdominal tergites III–V with medio-basal depression laterally bordered by more or less distinct oblique ridges (abdominal tergites III–VI with medio-basal depression laterally bordered by more or less distinct oblique ridges in *Philomyceta* and segment 2 of maxillary palpi comparatively slender in *Hesperoschema*) (Schillhammer 2004). The genus is currently subdivided into two subgenera, *Hesperosoma* Scheerpeltz, 1965 and *Paramichrotus* Naomi, 1982. The major differences separating the two subgenera are in the shape of aedeagus and the body colouration: with aedeagus symmetrical and fore body always with a certain amount of reddish colour on the elytra or pronotum in *Paramichrotus*, while the aedeagus is asymmetrical and fore-body with metallic tint throughout in *Hesperosoma* (Schillhammer 2015).

**Figure 1.**
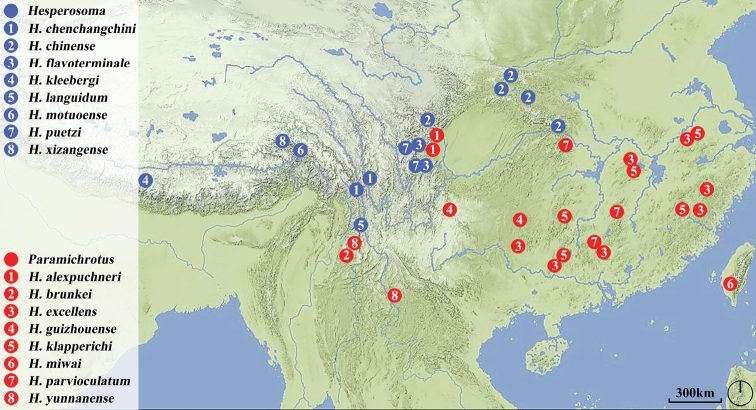
Distribution map of *Hesperosoma* species of China.

Up to now, nine species of the genus have been recorded from China. In this paper, five new species are described and two species are new country records for China. Thus, the total number of *Hesperosoma* in China is increased to 16. The members of the subgenus Hesperosoma are distributed in mountainous areas of southwest China, while the members of the subgenus Paramichrotus are widely distributed in southern China. (Fig. 1).

**Figures 2–8.**
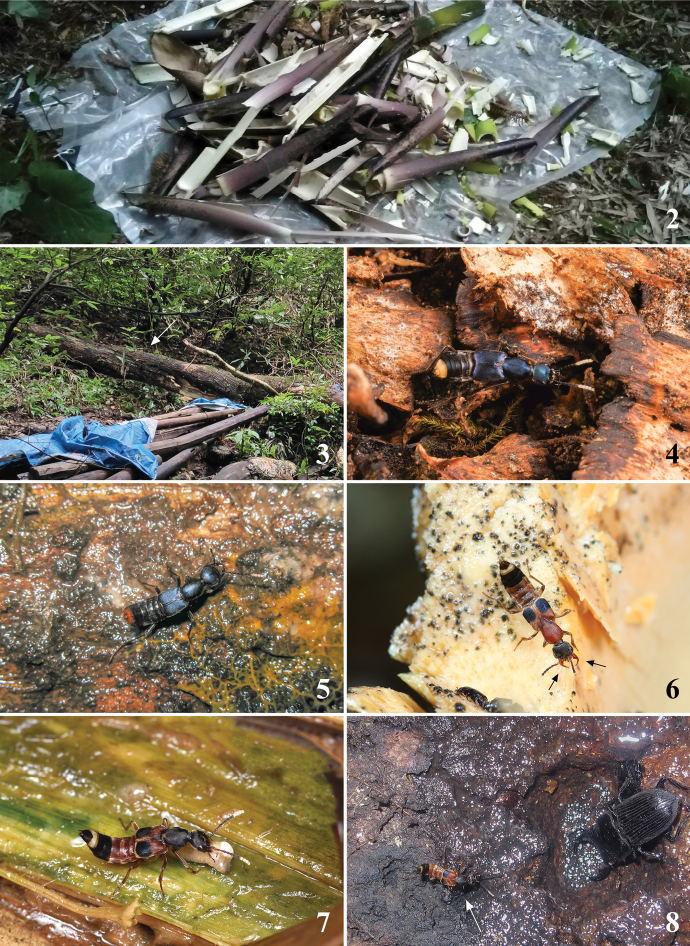
**2** rotten bamboo trap (Photo by Mr. Yu-Jie Cai from Guizhou, Leishan County, Xiannütang at 1 May 2021) **3** habitat of *H.flavoterminale* (Photo by Mr. Qin-Hao Zhao from Sichuan, Dujiangyan City, Mt Qingchengshan at 1 Aug. 2021) **4** living *H.flavoterminale* on decaying tree (Photo by Mr. Qin-Hao Zhao from Sichuan, Dujiangyan City, Mt Qingchengshan at 1 Aug 2021) **5** living *H.languidum* sp. nov. running on decaying tree with fungi during the night (Photo by Mr. Liang Tang from Yunnan, Lushui County, Yaojiapin at 21 Jun 2010) **6** living *H.excellens* on rotten bamboo (Photo by Mr. Liang Tang from Anhui, Tangkou Town, Jiulongpu at 1 Jul 2020) **7** living *H.guizhouense* hunting for maggots in rotten bamboo (Photo by Mr. Yu-Jie Cai from Guizhou, Leishan County, Xiannütang at 2 May 2021) **8** living *H.klapperichi* sucking tree sap (Photo by Mr. Zhong Peng from Guangxi, Jinxiu County, ‘16 km’ at 13 Jul 2014).

The species of the genus *Hesperosoma* can be usually found in undisturbed forests. Microhabitats include leaf litter, decaying logs and ground-based debris in woodlands with mixed shrub and grassland (Schillhammer, 2004, 2009, 2015, 2018; Hu et al. 2020). Based on our collecting experiences, the species of the subgenus Paramichrotus prefer rotten material, especially rotten bamboos, where they hunt for maggots (Figs 6, 7). Therefore, an efficient way to collect them is to set rotten bamboo traps (Fig. 2). Species of the *Paramichrotus* sometimes may also be found sucking sap around tree wounds (Fig. 8). A similar behaviour was previously described by Hu et al. (2020) on rotting stems of *Alocasiaodora* (G. Lodd.) Spach. Species of the subgenus Hesperosoma prefer decaying trees with fungi (Figs 3, 4). Members of both subgenera hide from light inside log or fungi crevices in daylight and they are usually spotted by searching logs in dense forests or at night (Fig. 5).

## ﻿Materials and methods

The specimens, examined in this paper, were collected by searching logs, sifting rotten bamboo or leaf litter and were euthanised with ethyl acetate. For examination of the genitalia, the last three abdominal segments were detached from the body after softening in hot water. The aedeagus or tergite X, together with other dissected pieces, was mounted in Euparal (Chroma Gesellschaft Schmidt, Koengen, Germany) on plastic slides. Photos of sexual characters were taken with a Canon G9 camera attached to an Olympus SZX 16 stereoscope; habitus photos were taken with a Canon macro photo lens MP–E 65 mm attached to a Canon EOS7D camera and stacked with Zerene Stacker.

The specimens treated in this study are deposited in the Department of Biology, Shanghai Normal University, P. R. China (**SHNU**) and Naturhistorisches Museum Wien, Austria (**NMW**).

### ﻿Body measurements are abbreviated as follows:

**BL** body length, measured from the anterior margin of the clypeus to the posterior margin of abdominal tergite X;

**EL** length of elytra, measured from humeral angle;

**EW** width of elytra at the widest point;

**EYL** length of eye;

**FL** fore-body length, measured from the anterior margin of the clypeus to the apex of the elytra (apicolateral angle);

**HL** length of head along the mid-line;

**HW** width of head including eyes;

**PL** length of pronotum along the mid-line;

**PW** width of pronotum at the widest point;

**TL** length of tempora.

### ﻿List of Chinese species

#### Subgenus Hesperosoma

*Hesperosomachenchangchini* sp. nov.

*Hesperosomachinense* Hayashi, 2002

*Hesperosomaflavoterminale* Schillhammer, 2004

*Hesperosomakleebergi* Schillhammer, 2009

*Hesperosomalanguidum* sp. nov.

*Hesperosomamotuoense* sp. nov.

*Hesperosomapuetzi* Schillhammer, 2004

*Hesperosomaxizangense* sp. nov.

#### Subgenus Paramichrotus

*Hesperosomaalexpuchneri* Schillhammer, 2009

*Hesperosomabrunkei* Schillhammer, 2015

*Hesperosomaexcellens* (Bernhauer, 1939)

*Hesperosomaguizhouense* Schillhammer, 2018

*Hesperosomaklapperichi* Schillhammer, 2004

*Hesperosomamiwai* (Bernhauer, 1943)

*Hesperosomaparvioculatum* sp. nov.

*Hesperosomayunnanense* Schillhammer, 2009

### ﻿Key to species of *Hesperosoma* from China

**Table:** 

1	Elytra entirely dark, always with distinct metallic blue or greenish-blue lustre; aedeagus at least weakly asymmetrical (subgenus Hesperosoma)	**2**
–	Elytra not metallic, with at least some reddish colouration, usually extensive, rarely reduced to shoulders; aedeagus symmetrical (subgenus Paramichrotus)	**9**
2	Fore-body very shiny; punctation less dense, most punctures simple, widely separated	***H.motuoense* sp. nov. China (Xizang)**
–	Fore-body rather opaque due to very dense punctation; punctures subumbilicate, almost contiguous	**3**
3	First three or four visible abdominal segments reddish	**4**
–	First five visible abdominal segments black	**5**
4	Four apical segments of antennae creamy white; abdominal segment VIII entirely reddish-yellow	***H.chenchangchini* sp. nov. China (Yunnan)**
–	Five apical segments of antennae creamy white; abdominal segment VIII blackish-brown with base of the tergite widely yellowish	***H.chinense* China (Shaanxi, Hubei, Sichuan)**
5	Five apical segments of antennae creamy white	**6**
–	Four apical segments of antennae creamy white	**7**
6	Pronotum deep metallic blue, relatively weak build; pubescence of first three visible tergites entirely golden	***H.flavoterminale* China (Sichuan)**
–	Pronotum brighter metallic blue, more robust build; pubescence of first three visible tergites entirely black	***H.kleebergi* Nepal, China (Xizang)**
7	Head 1.29 times as wide as long; frons with anterior margin emarginate (Fig. 9); mandibles relatively darker	***H.xizangense* sp. nov. China (Xizang)**
–	Head 1.14–1.22 times as wide as long; frons with anterior margin nearly straight (Fig. 8); mandibles relatively lighter	**8**
8	TL/EYL: 1.78; head, pronotum and elytra with dense and faint punctation (Fig. 10); first three visible abdominal tergites with shallower punctures in basal half	***H.languidum* sp. nov. China (Yunnan)**
–	TL/EYL: 1.47–1.48; head, pronotum and elytra with dense and coarse punctation (Fig. 8); first three visible abdominal tergites with pit-like punctures in basal half	***H.puetzi* China (Sichuan)**
9	Pronotum black, procoxae partly and lateral parts of mesoventrite dark brown to black	**10**
–	Pronotum entirely rufous, rarely with darker markings, procoxae and mesoventrite reddish	**15**
10	Elytra with black marking occupying more than posterior half of each elytron, with elevated, reddish sutural stripe connects with black marking for at least half of its length	**11**
–	Elytra with black marking occupying at most posterior half of each elytron, usually less, marking not reaching elevated reddish sutural stripe or only narrowly, at the postero-sutural angle	**12**
11	TL/EYL: 1.18–1.29; head 1.26–1.40 times as wide as long	***H.yunnanense* China (Yunnan)**
–	TL/EYL: 1.35–1.39; head 1.63 times as wide as long	***H.parvioculatum* sp. nov. China (Hubei, Hunan)**
12	All tibiae predominantly black	***H.miwai* China (Taiwan)**
–	Tibiae predominantly reddish or bicoloured	**13**
13	Paramere with acutely pointed apex	***H.alexpuchneri* China (Sichuan)**
–	Paramere bilobed	**14**
14	Paramere (Fig. 75) with relatively deeper medio-apical emargination	***H.guizhouense* China (Guizhou)**
–	Paramere (Fig. 81) with relatively shallower medio-apical emargination	***H.klapperichi* China (Anhui, Fujian, Guangxi, Hubei, Hunan)**
15	Basal antennomeres 3–4 bright reddish; maxillary palpi entirely reddish	***H.excellens* China (Anhui, Fujian, Guangdong, Guangxi, Hubei, Hunan, Zhejiang)**
–	Basal antennomeres 3–4 at least partly blackish; segments 2 and 3 of maxillary palpi reddish-brown	***H.brunkei* Laos, Myanmar, China (Yunnan)**

### ﻿Taxonomy

#### Subgenus Hesperosoma

##### ﻿ Hesperosoma (s.str.) chenchangchini sp. nov.

Taxon classificationAnimaliaColeopteraStaphylinidae

52F876D3-27E2-5A42-946B-DCE8B10B611A

http://zoobank.org/D92FFB10-ADD6-4FC8-A13A-3F283D80125E

Figures 9 , 13–18

###### Material examined.

***Holotype*.** China – **Yunnan Prov.** • ♂; glued on a card with labels as follows: “China: Yunnan Deqin County, Nagu Vill; alt. 2250 m; 11 Jul 2010; Wen-Xuan Bi leg.” “Holotype / Hesperosoma(s.str.)chenchangchini / Cai, Tang & Schillhammer” [red handwritten label]; SHNU. ***Paratypes*.** China – **Yunnan Prov.** • 2♂ ♂, 1 ♀; Gongshan County, Heiwadi; alt. 2000 m; 07 Jul 2009; Jian-Qing Zhu leg.; SHNU.

###### Description.

Measurements of male: BL: 12.17–13.87 mm, FL: 6.95–7.63 mm. HL: 1.89–2.07 mm, HW: 2.41–2.79 mm, EYL: 0.65–0.77 mm, TL: 0.96–1.12 mm, PL: 2.30–2.68 mm, PW: 2.00–2.23 mm, EL: 3.13–3.44 mm, EW: 3.06–3.51 mm. HW/HL: 1.24–1.35, TL/EYL: 1.41–1.48, PL/PW: 1.14–1.20, EL/EW: 0.98–1.02.

**Measurements of female**: BL: 12.73 mm, FL: 7.06 mm. HL: 1.89 mm, HW: 2.34 mm, EYL: 0.68 mm, TL: 0.90 mm, PL: 2.38 mm, PW: 2.07 mm, EL: 3.17 mm, EW: 3.28 mm. HW/HL: 1.24, TL/EYL: 1.32, PL/PW: 1.15, EL/EW: 0.97.

Head, pronotum and elytra metallic dark blue to violaceous blue; abdomen with segments III–V reddish, VI black with anterior margin narrowly reddish, VII black with posterior margin broadly reddish-yellow, segments VIII and X entirely reddish-yellow, segment IX reddish; antennae black, base and apex of segment 1 and 2 reddish, segments 8–11 creamy white; mandibles dark brown, medial margin and distal portion of mandible dark reddish-brown; maxillary and labial palpi deep black, last segments sometimes slightly paler brownish.

**Figures 9–12.**
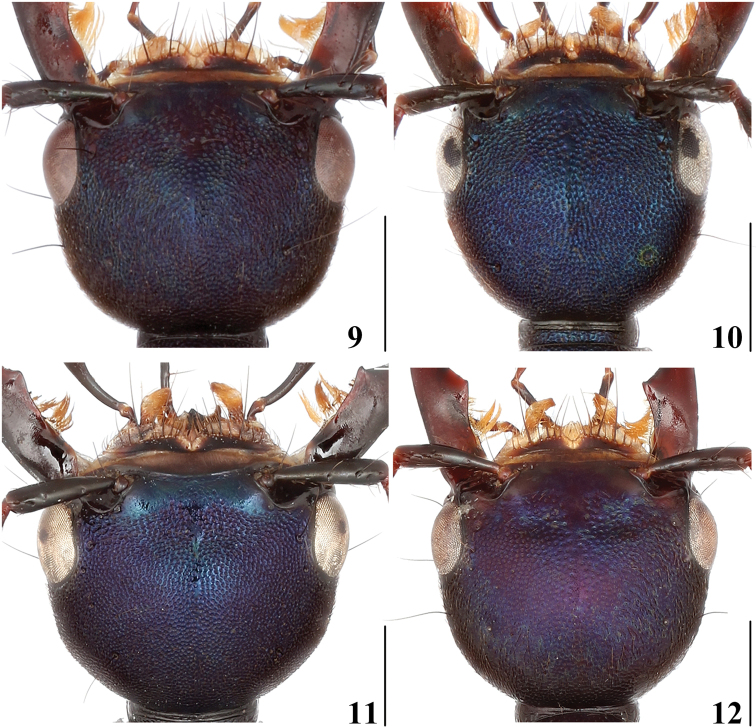
Head of species of subgenus Hesperosoma**9***H.chenchangchini* sp. nov. **10***H.puetzi***11***H.xizangense* sp. nov. **12***H.languidum* sp. nov. Scale bars: 1 mm.

Head (Fig. 9) 1.24–1.35 times as wide as long, rounded trapezoid, tempora narrowed towards neck in regular arc, eyes moderately protruding; surface with dense and coarse punctation, mostly contiguous; frons impunctate; with short, weakly delimited impunctate mid-line, extending from frons to about half of mid-length; antennae with segments 4–8 markedly oblong, segments 9 and 10 about as long as wide.

Pronotum 1.14–1.20 times as long as wide, widest at level of large lateral seta, narrowed towards base in wide, but shallow concave arc; surface as densely and coarsely punctate as on head, with indistinct, short impunctate mid-line in posterior half; scutellum with dense and pit-like punctation, interstices forming small transverse rugae.

Elytra 0.97–1.02 times as long as wide, exceedingly densely, coarsely punctate, punctures almost contiguous.

Abdominal tergites III–V with basal transverse depression, punctation of abdominal tergites IV–V moderate and sparse at base, abdominal tergite III impunctate at base; posterior halves of abdominal tergites III–V and entire surface of remaining tergites with very fine and dense punctuation.

**Male.** Protarsomeres 1–4 moderately dilated, heart-shaped; sternite VII with patch of yellow setae on median portion and posterior margin broadly emarginate at middle; sternite VIII with posterior margin broadly emarginate at middle; aedeagus (Figs 15–17) with median lobe and paramere slightly asymmetrical, paramere (Fig. 17) shorter than median lobe and slightly bent to left side in ventral view.

**Female.** Tergite X (Fig. 18) slightly asymmetrical with posterior margin projecting at middle.

**Figures 13–18.**
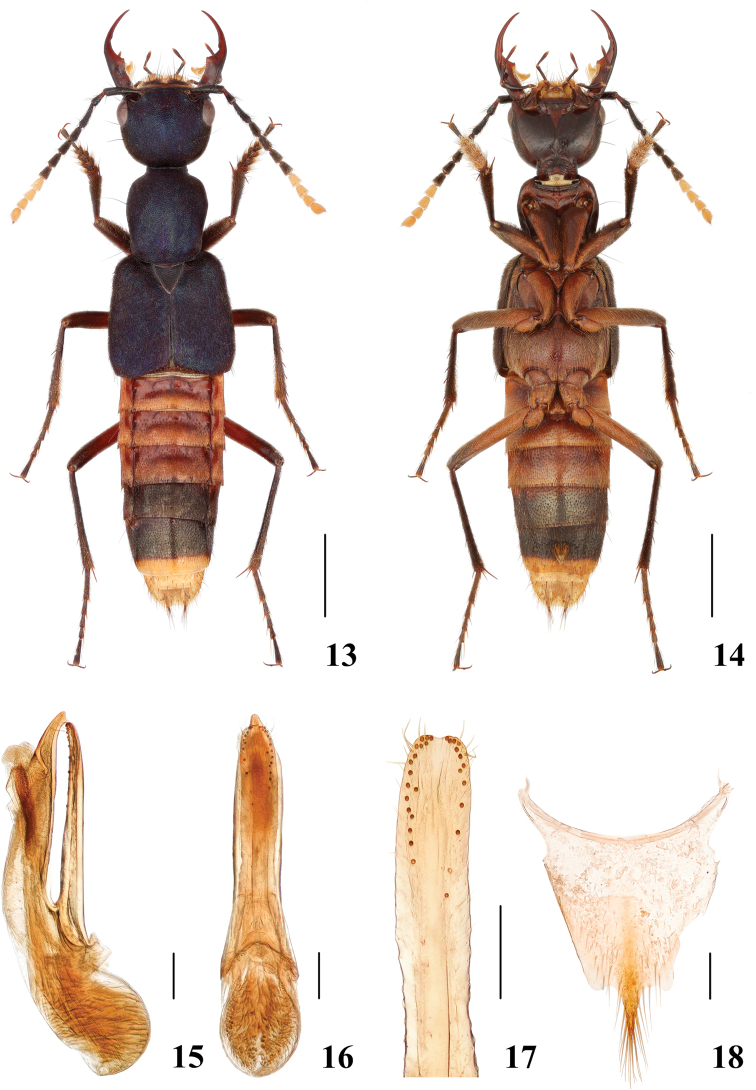
*Hesperosomachenchangchini* sp. nov. **13–14** habitus **15–17** aedeagus, lateral (**15**) and ventral (**16**) views, paramere (**17**) **18** female abdominal tergite X. Scale bars: 2 mm (**13–14**), 0.2 mm (**15–18**).

###### Etymology.

This species is named in honour of Mr. Chang-Chin Chen who donates lots of staphylinid specimens to the SHNU, including the holotype of the new species.

###### Distribution.

China (Yunnan).

###### Diagnosis.

Amongst the species of the nominal subgenus with reddish abdomen, *H.malaisei* from Myanmar, *H.chinense* (Shaanxi, Hubei, Sichuan) and *H.rufomarginatum* from Vietnam, the new species may be easily recognised by four outer segments of antennae creamy white (five outer segments of antennae creamy white in *H.chinense* and *H.malaisei*), entirely metallic dark blue to violaceous blue elytra (reddish suture and shoulders of elytra in *H.rufomarginatum*).

##### ﻿ Hesperosoma (s.str.) chinense

Taxon classificationAnimaliaColeopteraStaphylinidae

Hayashi, 2002

23E0ED86-C56E-53CC-BFBB-1602ED9254F3

Figures 19–24

Hesperosoma chinense Hayashi 2002: 175; Schillhammer 2004: 256; Schillhammer 2009: 84

###### Material examined.

China – **Shaanxi Prov.** • 1♂; Zhouzhi County, Houzhenzi, Qinling, Qinlingliangxia; 33°48'96"N, 107°44'48"E; alt. 2018 m; 7 May 2008; Hao Huang & Wang Xu leg.; SHNU • 1♂; Zhouzhi County, Houzhenzi, Qinling, Qinlingliangxia; 33°48'97"N, 107°44'48"E; alt. 1820 m; 18 May 2008; Hao Huang & Wang Xu leg.; SHNU • 1♂, 1♀; Zhouzhi County, Houzhenzi, Qinling, West Sangongli Gou; 33°50'613"N, 107°48'524"E; alt. 1336 m; 17–19 May 2008; Hao Huang & Wang Xu leg.; SHNU • 1♂; Mei County, Taibai-Shan, Kaitianguan; 34°00'69"N, 107°51'41"E; alt. 1850 m; 22–23 May 2008; Hao Huang & Wang Xu leg.; SHNU • 2♂♂, 5♀♀; Ankang City, Ningshan County, Huoditang; 33°44'N, 108°45'E; alt. 1590 m; 19 Jul 2015; Yi-Zhou Liu leg.; SHNU • 1♂; Hanzhong City Mian County; 33°10'24"N, 106°40'13"E; alt. 1800 m; 24 Jun 2020; W-X Bi leg.; SHNU • 9♀♀; Ankang City, Ningshan County, Huodigou; 33°46'N, 108°44'E; alt. 1540 m; 18 Jul 2015; Yi-Zhou Liu leg.; SHNU. – **Sichuan Prov.** • 1♂; Aba Pre, Li County; alt. 1800–2300 m; 29 Jun 2015; Hao Huang leg.; SHNU.

###### Measurements.

**Male**: BL: 10.45–14.56 mm, FL: 6.06–8.11 mm. HL: 1.72–2.11 mm, HW: 2.00–2.66 mm, EYL: 0.61–0.71 mm, TL: 0.83–1.11 mm, PL: 2.11–2.63 mm, PW: 1.77–2.22 mm, EL: 2.72–3.43 mm, EW: 2.78–3.33 mm. HW/HL: 1.16–1.26, TL/EYL: 1.36–1.68, PL/PW: 1.16–1.23, EL/EW: 0.98–1.03.

**Female**: BL: 13.06–16.79 mm, FL: 6.95–8.22 mm. HL: 1.94–2.24 mm, HW: 2.27–2.55 mm, EYL: 0.61–0.72 mm, TL: 0.94–1.11 mm, PL: 2.39–2.72 mm, PW: 2.00–2.33 mm, EL: 3.11–3.61 mm, EW: 3.11–3.78 mm. HW/HL: 1.14–1.21, TL/EYL: 1.42–1.64, PL/PW: 1.14–1.20, EL/EW: 0.96–1.03.

###### Distribution.

China (Hubei, Shaanxi and Sichuan).

###### Diagnosis.

Amongst the species of the subgenus with reddish abdomen, *H.chinense* may be readily recognised from *H.chenchangchini* sp. nov. and *H.rufomarginatum* by five outer segments of antennae creamy white (four outer segments of antennae creamy white in *H.chenchangchini* sp. nov. and *H.rufomarginatum*); abdomen reddish in basal four visible segments, segment VIII blackish-brown with base of the tergite broadly yellowish, segment IX blackish-brown (abdomen reddish in basal three visible segments, segments VIII and IX pale yellow in *H.malaisei*).

**Figures 19–24.**
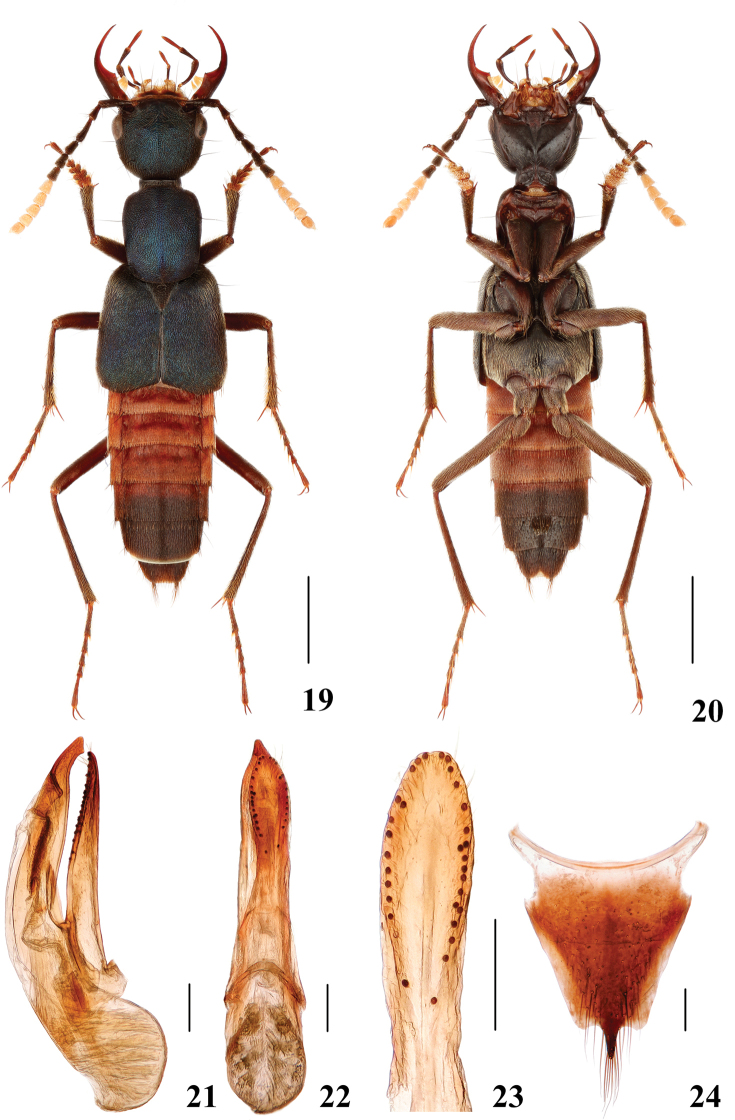
*Hesperosomachinense***19–20** habitus **21–23** aedeagus, lateral (**21**) and ventral (**22**) views, paramere (**23**) **24** female abdominal tergite X. Scale bars: 2 mm (**19–20**), 0.2 mm (**21–24**).

##### ﻿ Hesperosoma (s.str.) flavoterminale

Taxon classificationAnimaliaColeopteraStaphylinidae

Schillhammer, 2004

74A4FC65-F82C-5889-9B6C-7052646FD7B2

Figures 25–30

Hesperosoma flavoterminale Schillhammer 2004: 257; Schillhammer 2009: 85

###### Material examined.

China – **Sichuan Prov.** • 1♂; Shimian County, Liziping, Zima Village; 28°59'N, 102°16'E; alt. 1800 m; 16 Aug 2012; Peng, Dai & Yin leg.; SHNU • 1♂; Hailuogou, Qingshibangou; alt. 2200 m; 20 Jul 2011; Hao Huang leg.; SHNU • 1♂, 5♀♀; Baoxing County, Fengtongzhai nat. cons., Dashuigou station; 30.57247N, 102.88260E; alt. 1569 m; 29 Jun 2013; Li leg.; by pitfall trap, broadleaf forest; SHNU.

###### Measurements.

**Male**: BL: 12.62–14.13 mm, FL: 6.85–7.65 mm. HL: 1.89–2.13 mm, HW: 2.32–2.66 mm, EYL: 0.68–0.74 mm, TL: 0.94–1.08 mm, PL: 2.29–2.54 mm, PW: 1.92–2.23 mm, EL: 3.03–3.40 mm, EW: 3.03–3.49 mm. HW/HL: 1.23–1.26, TL/EYL: 1.41–1.46, PL/PW: 1.14–1.23, EL/EW: 0.97–1.04.

**Female**: BL: 13.33–15.82 mm, FL: 7.29–7.84 mm. HL: 1.98–2.17 mm, HW: 2.35–2.54 mm, EYL: 0.71–0.77 mm, TL: 0.96–1.05 mm, PL: 2.48–2.66 mm, PW: 2.10–2.23 mm, EL: 3.32–3.51 mm, EW: 3.40–3.62 mm. HW/HL: 1.16–1.19, TL/EYL: 1.25–1.44, PL/PW: 1.14–1.21, EL/EW: 0.97–0.98.

###### Female characters.

Tergite X (Fig. 30) with posterior margin projecting at middle.

###### Distribution.

China (Sichuan).

###### Diagnosis.

Externally, the species is very similar to *H.kleebergi*, but differs mainly in relatively weak build, dark metallic colour, larger eyes, narrow reddish-yellow apical margin of abdominal segment VII and entirely golden pubescence of first three visible tergites (pubescence of first three visible tergites entirely black in *H.kleebergi*).

**Figures 25–30.**
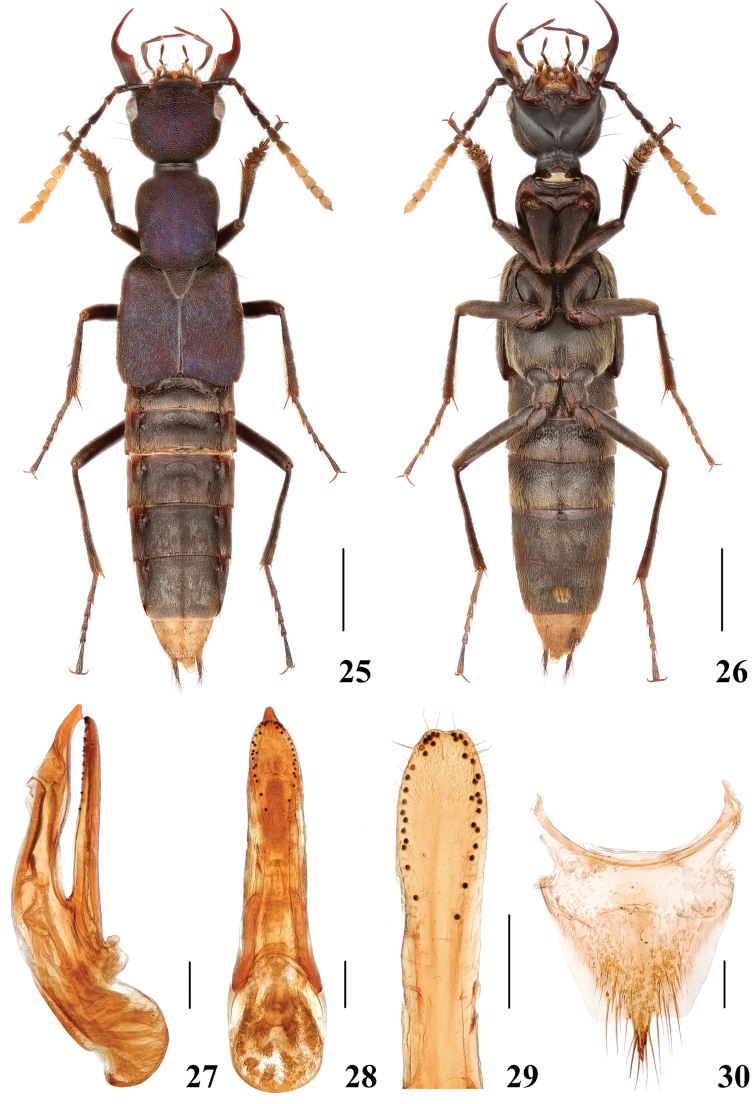
*Hesperosomaflavoterminale***25–26** habitus **27–29** aedeagus, lateral (**27**) and ventral (**28**) views, paramere (**29**) **30** female abdominal tergite X. Scale bars: 2 mm (**25–26**), 0.2 mm (**27–30**).

##### ﻿ Hesperosoma (s.str.) kleebergi

Taxon classificationAnimaliaColeopteraStaphylinidae

Schillhammer, 2009

8462B9C1-A80F-58AC-8EC3-712B7072838F

Figures 31–35

Hesperosoma kleebergi Schillhammer, 2009: 85

###### Material examined.

China – **Xizang Prov.** • 1♂; Xizang A. R., Nielamu County, Lixin Vill.; alt. 2600 m; 24 Jul 2010; Wen-Xuan Bi leg.; SHNU.

###### Measurements.

**Male**: BL: 13.19 mm, FL: 7.48 mm. HL: 2.07 mm, HW: 2.60 mm, EYL: 0.71 mm, TL: 1.05 mm, PL: 2.45 mm, PW: 2.15 mm, EL: 3.51 mm, EW: 3.40 mm. HW/HL: 1.26, TL/EYL: 1.48, PL/PW: 1.14, EL/EW: 1.03.

###### Distribution.

China (Xizang) and Nepal. New to China.

###### Remarks.

The collecting locality of the male specimen is about 200 km away from the type locality in Nepal. The specimen fits the original description in all characters. New record for China.

**Figures 31–35.**
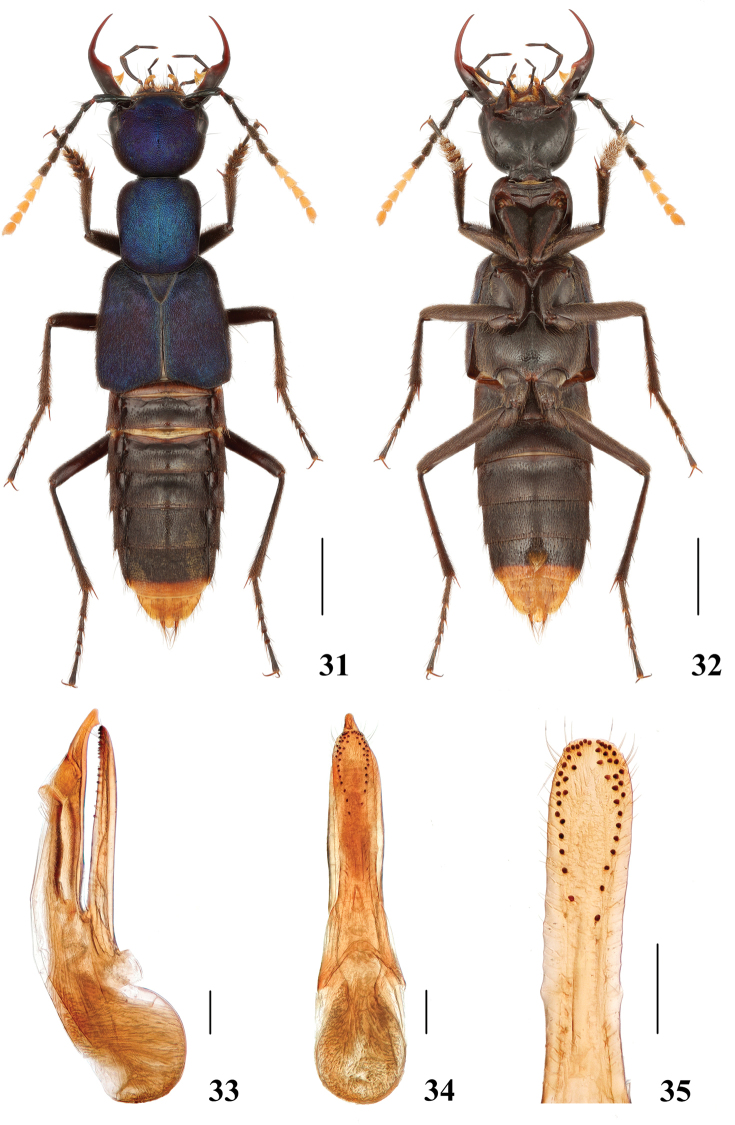
*Hesperosomakleebergi***31–32** habitus **33–35** aedeagus, lateral (**33**) and ventral (**34**) views, paramere (**35**). Scale bars: 2 mm (**31–32**), 0.2 mm (**33–35**).

##### ﻿ Hesperosoma (s.str.) languidum sp. nov.

Taxon classificationAnimaliaColeopteraStaphylinidae

889F029C-1AEE-5886-89E8-6412C287FBC6

http://zoobank.org/4E16817A-3DFF-42A4-8519-A49E88436839

Figures 12 , 36–40

###### Material examined.

***Holotype*.** China – **Yunnan Prov.** • ♂; glued on a card with labels as follows: “China: Yunnan, Lushui County, Yaojiapin; alt. 2540 m; 21 Jun 2010; Liang Tang leg.” “Holotype / Hesperosoma(s.str.)languidum / Cai, Tang & Schillhammer” [red handwritten label]; SHNU.

###### Description.

**Measurements of male**: BL: 15.63 mm, FL: 7.48 mm. HL: 2.19 mm, HW: 2.68 mm, EYL: 0.68 mm, TL: 1.21 mm, PL: 2.72 mm, PW: 2.27 mm, EL: 3.06 mm, EW: 3.05 mm. HW/HL: 1.22, TL/EYL: 1.78, PL/PW: 1.20, EL/EW: 1.00.

Head and pronotum metallic violaceous blue to deep blue, elytra dark metallic violaceous blue, usually a bit more opaque than head and pronotum; abdomen with segments III–VI dark red, segment VII dark red with posterior margin broadly reddish-yellow, segments VIII and X entirely reddish-yellow, segment IX reddish-yellow with apical third of latter blackish; antennae black, base and apex of segments 1 and 2 and base of segment 3 reddish, four outer segments creamy white; mandibles dark reddish-brown; maxillary palpi with segments I–III black, segment IV brown, labial palpi with segments I and II black, segment III brown.

**Figures 36–40.**
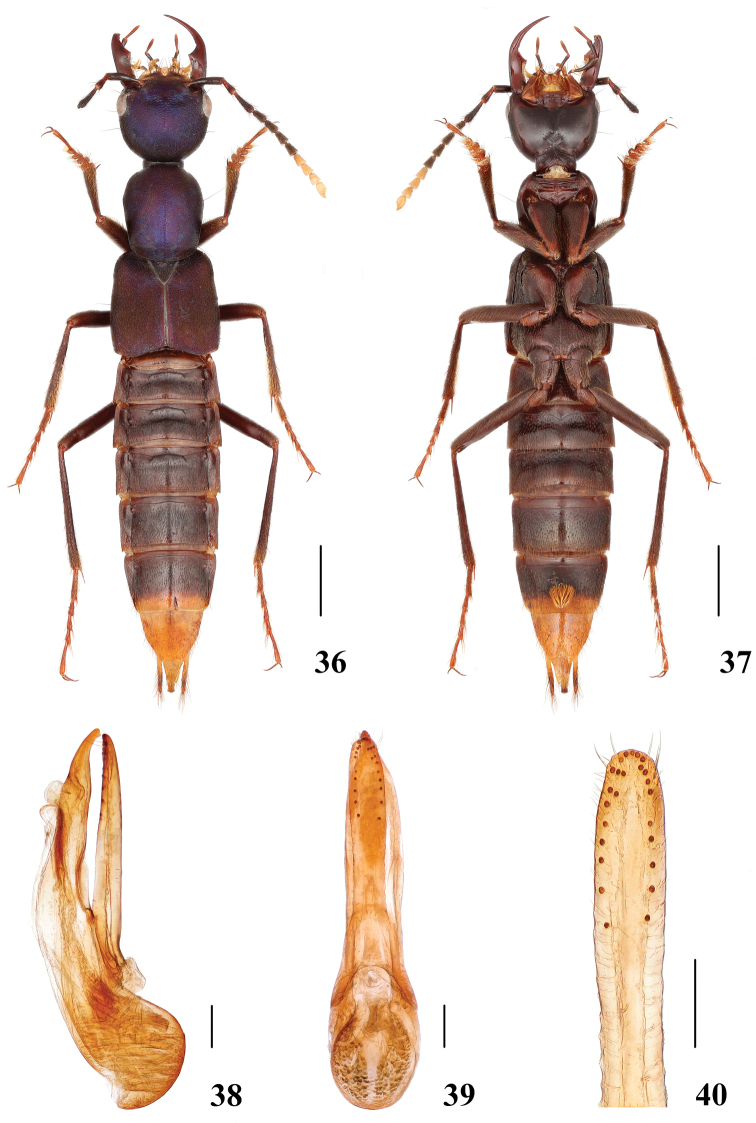
*Hesperosomalanguidum* sp. nov. **36–37** habitus **38–40** aedeagus, lateral (**38**) and ventral (**39**) views, paramere (**40**). Scale bars: 2 mm (**36–37**), 0.2 mm (**38–40**).

Head (Fig. 12) 1.22 times as wide as long, rounded trapezoid, tempora regularly convex, eyes moderately protruding; surface with dense and shallow punctation, mostly contiguous; frons impunctate; with short, weakly delimited impunctate mid-line, extending from impunctate frons to about half of mid-length; antennae with segments 4–8 markedly oblong, segment 9 slightly oblong.

Pronotum 1.20 times as long as wide, slender, widest at level of large lateral seta, narrowed towards base in wide, but shallow concave arc; surface as densely and shallowly punctate as on head, with indistinct, short impunctate mid-line in posterior third; scutellum with dense and shallow punctation, interstices forming small transverse rugae.

Elytra as long as wide, exceedingly densely, shallowly punctate, punctures almost contiguous.

Abdominal tergites III–V with basal transverse depression, punctation of abdominal tergites III–V feeble at base; posterior halves of abdominal tergites III–V and entire surface of remaining tergites with very fine and dense punctation.

**Male.** Protarsomeres 1–4 moderately dilated, heart-shaped; sternite VII with patch of long bright yellow setae on median portion; sternite VIII with posterior margin emarginate at middle; aedeagus (Figs 38–40) with median lobe and paramere slightly asymmetrical, paramere (Fig. 40) relatively shorter than median lobe, aedeagus very similar to that of *H.puetzi*, but paramere narrower and apex of the median lobe markedly slender in lateral view.

**Female.** Unknown.

###### Etymology.

The specific epithet refers to the shallow punctation of the new species

###### Distribution.

China (Yunnan).

###### Diagnosis.

The new species is very similar to *H.puetzi* (Sichuan), but can be easily distinguished from it by smaller eyes with TL/EYL about 1.70 (1.47 in *H.puetzi*); head, pronotum and elytra with dense and shallow punctation (punctation in *H.puetzi* dense and coarse); abdominal tergites III–V without pit-like punctures in basal half. In appearance, it is also similar to *H.flavoterminale* (Sichuan), *H.tarasovi* from Laos and *H.kleebergi* from China (Xizang) and Nepal, but can be distinguished from *H.flavoterminale* and *H.kleebergi* by the antennal segments VIII–XI being creamy white (segments VII–XI creamy white in *H.flavoterminale* and *H.kleebergi*); and from *H.tarasovi* by the narrower head.

##### ﻿ Hesperosoma (s.str.) motuoense sp. nov.

Taxon classificationAnimaliaColeopteraStaphylinidae

F7CDA45E-6F9D-5638-90DE-B1D32B84F60B

http://zoobank.org/43DC52F6-AD98-4328-8E3B-93D67DAC68F1

Figures 41–46

###### Material examined.

***Holotype*.** China – **Xizang Prov.** • ♂; glued on a card with labels as follows: “China: Xizang A. R., Motuo County, Hanmi; alt. 2100 m; 23 Aug 2011; Wen-Xuan Bi leg.” “Holotype / Hesperosoma(s.str.)motuoense / Cai, Tang & Schillhammer” [red handwritten label]; SHNU. ***Paratypes*.** China – **Xizang Prov.** • 3♂♂; Motuo County, 80k; alt. 2100 m; 24 Aug 2011; Wen-Xuan Bi leg.; SHNU, NMW • 2♀♀; same locality, but 11 Aug 2013, Wen-Xuan Bi leg.; SHNU, NMW • 2♂♂; same locality, but 11 Aug 2013; Wen-Xuan Bi leg.; SHNU • 1♀; same locality, but 22 Jul 2013; Wen-Xuan Bi leg.; SHNU • 1♂; Zhucun-Bangxin; alt. 1850 m; 26 Aug 2013; Wen-Xuan Bi leg.; SHNU.

###### Measurements.

**Male**: BL: 10.54–14.54 mm, FL: 6.12–7.25 mm. HL: 1.77–2.07 mm, HW: 2.11–2.68 mm, EYL: 0.64–0.75 mm, TL: 0.83–1.05 mm, PL: 2.00–2.45 mm, PW: 1.77–2.19 mm, EL: 2.75–3.21 mm, EW: 2.75–3.28 mm. HW/HL: 1.19–1.34, TL/EYL: 1.25–1.44, PL/PW: 1.11–1.15, EL/EW: 0.94–1.00.

**Female**: BL: 13.23–15.49 mm, FL: 7.10–7.40 mm. HL: 1.96–2.07 mm, HW: 2.34–2.45 mm, EYL: 0.75–0.79 mm, TL: 0.94–1.02 mm, PL: 2.41–2.45 mm, PW: 2.07–2.15 mm, EL: 3.17–3.40 mm, EW: 3.25–3.40 mm. HW/HL: 1.16–1.20, TL/EYL: 1.25–1.29, PL/PW: 1.14–1.18, EL/EW: 0.96–1.00.

Head and pronotum brilliant metallic green to bluish-green, elytra brighter metallic green, bluish-green at shoulders, along sides and at posterolateral angles; abdomen with segments III–V reddish (in one specimen, abdomen with segments III–V reddish, medio-posterior portion darkened on segments IV and V), segments VI–VII dark brown, but segment VII with apical portion broadly yellow, segments VIII–IX entirely pale yellow; antennae with segments 1–6 black, base of segment 2 reddish, segments 7–11 creamy white; mandibles reddish with narrowly darkened medial and lateral margins; palpi dark reddish-brown with paler reddish tips; legs reddish-brown.

Head 1.16–1.34 times as wide as long, rounded trapezoid, tempora narrowed behind eyes; surface with moderately dense, simple punctation, punctures separated by about 1–2 puncture diameters in transverse direction; narrow anterior portion of frons impunctate; narrow impunctate mid-line extending from frons posteriad to mid-length; pubescence brownish; antennae with segments 4–7 markedly oblong, segments 8 and 9 slightly oblong, segment 10 about as long as wide.

Pronotum 1.11–1.18 times as long as wide, widest at about level of large lateral seta, narrowed towards base in distinct concave arc; punctation of surface similar to that of head, with narrow impunctate mid-line; scutellum densely furnished with pit-like punctures, but punctures, although almost contiguous, well isolated.

Elytra 0.94–1.00 times as long as wide, surface slightly uneven, with distinct depression between shoulders, scutellum and apical margin of elytra, along suture slightly elevated; punctation dense, but punctures not contiguous, separated by less than a puncture diameter in transverse direction; pubescence yellow, long and dense along suture and posterior elytral margin.

Abdominal tergites III–V with basal transverse depression; punctation of abdominal tergites III–V pit-like at base, gradually becoming finer towards apical margin, pit-like punctures occupying more than basal half on tergite III, about basal half on tergite IV and about basal third on tergite V; abdominal tergites VI–VIII with punctures similar in size, interstices smooth.

**Male.** Protarsomeres 1–4 moderately dilated, heart-shaped; sternite VII with patch of long yellow setae on median portion, posterior margin broadly emarginate at middle; sternite VIII with posterior margin emarginate at middle; aedeagus (Figs 43–45) with median lobe and paramere slightly asymmetrical, paramere (Fig. 45) shorter than median lobe and medio-apically emarginate.

**Figures 41–46.**
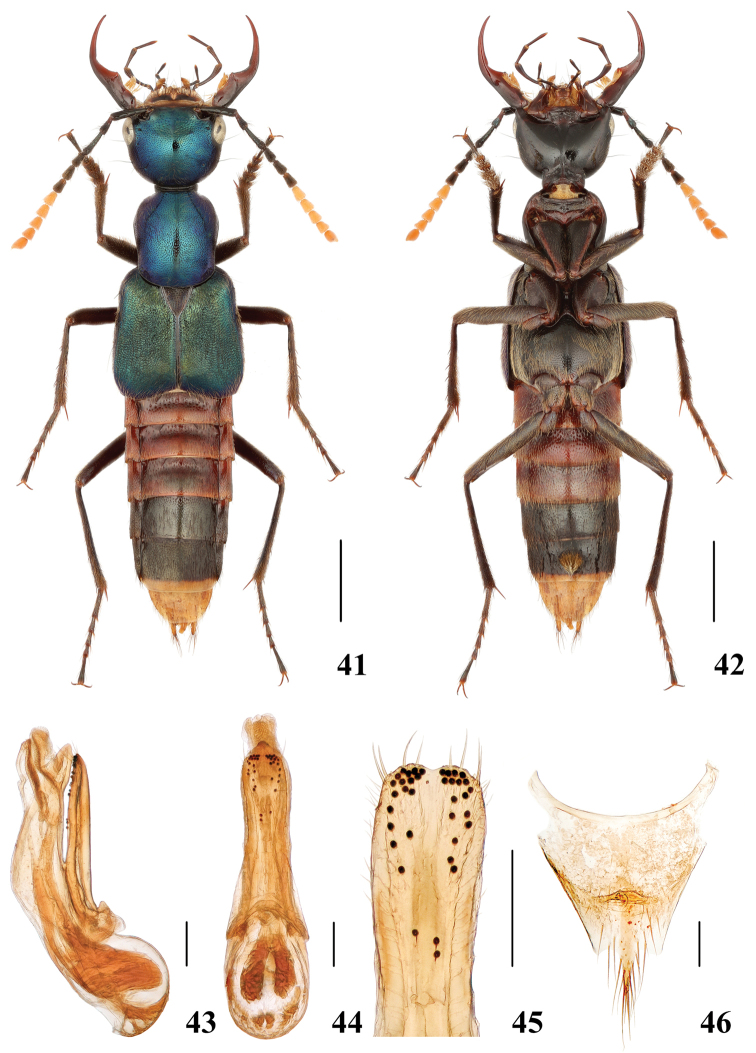
*Hesperosomamotuoense* sp. nov. **41–42** habitus **43–45** aedeagus, lateral (**43**) and ventral (**44**) views, paramere (**45**) **46** female abdominal tergite X. Scale bars: 2 mm (**41–42**), 0.2 mm (**43–46**).

**Female.** Tergite X (Fig. 46) slightly asymmetrical with posterior margin projecting at middle.

###### Etymology.

The species is named after the type locality.

###### Distribution.

China (Xizang).

###### Diagnosis.

Externally, the species hardly differs from *H.mishmiense* Schillhammer, 2004 from India, but may be distinguished by the shape of the aedeagus (Figs 43–45): median lobe (lateral view) in *H.motuoense* sp. nov. broader than in *H.mishmiense*, paramere in *H.motuoense* sp. nov. with shallower medio-apical emargination.

##### ﻿ Hesperosoma (s.str.) puetzi

Taxon classificationAnimaliaColeopteraStaphylinidae

Schillhammer, 2004

3D94A74F-E319-571C-AAA1-B247B9D995D7

Figures 10 , 47–52

Hesperosoma puetzi Schillhammer 2004: 256; Schillhammer 2009: 84

###### Material examined.

China – **Sichuan Prov.** • 1♂; Luding County, Hailuogou, Caohaizi; alt. 2780 m; 28 Jun 2009; Li-Zhen Li leg.; SHNU • 1♂; Shimian County, Liziping, Yele Dam; 28°55'N, 102°13'E; alt. 2600 m; 15 Jul 2012; Peng, Dai & Yin leg.; SHNU • 1♀; Luding County, Hailuogou, Qingshibangou; alt. 2200 m; 20 Jul 2011; Hao Huang leg.; SHNU.

###### Measurements.

**Male**: BL: 12.83–13.20 mm, FL: 6.61–6.69 mm. HL: 1.89–1.92 mm, HW: 2.15–2.22 mm, EYL: 0.64–0.65 mm, TL: 0.94–0.96 mm, PL: 2.30–2.32 mm, PW: 1.92 mm, EL: 2.94–3.04 mm, EW: 3.02–3.07 mm. HW/HL: 1.14–1.16, TL/EYL: 1.47–1.48, PL/PW: 1.20–1.21, EL/EW: 0.97–0.99.

**Female**: BL: 12.30 mm, FL: 7.07 mm. HL: 1.92 mm, HW: 2.29 mm, EYL: 0.74 mm, TL: 0.93 mm, PL: 2.45 mm, PW: 1.98 mm, EL: 3.04 mm, EW: 3.25 mm. HW/HL: 1.19, TL/EYL: 1.26, PL/PW: 1.24, EL/EW: 0.94.

###### Distribution.

China (Sichuan).

###### Diagnosis.

Amongst the species of the nominal subgenus with four outer segments of antennae creamy white, *H.puetzi* may be easily recognised by the narrower head (HW/HL: 1.14–1.16 in *H.puetzi*, HW/HL: 1.22 in *H.languidum*, HW/HL: 1.29 in *H.xizangense*, HW/HL: 1.24–1.35 in *H.chenchangchini*) and other differences (see diagnoses in Hesperosoma(s.str.)xizangense sp. nov. and Hesperosoma(s.str.)languidum sp. nov.).

**Figures 47–52.**
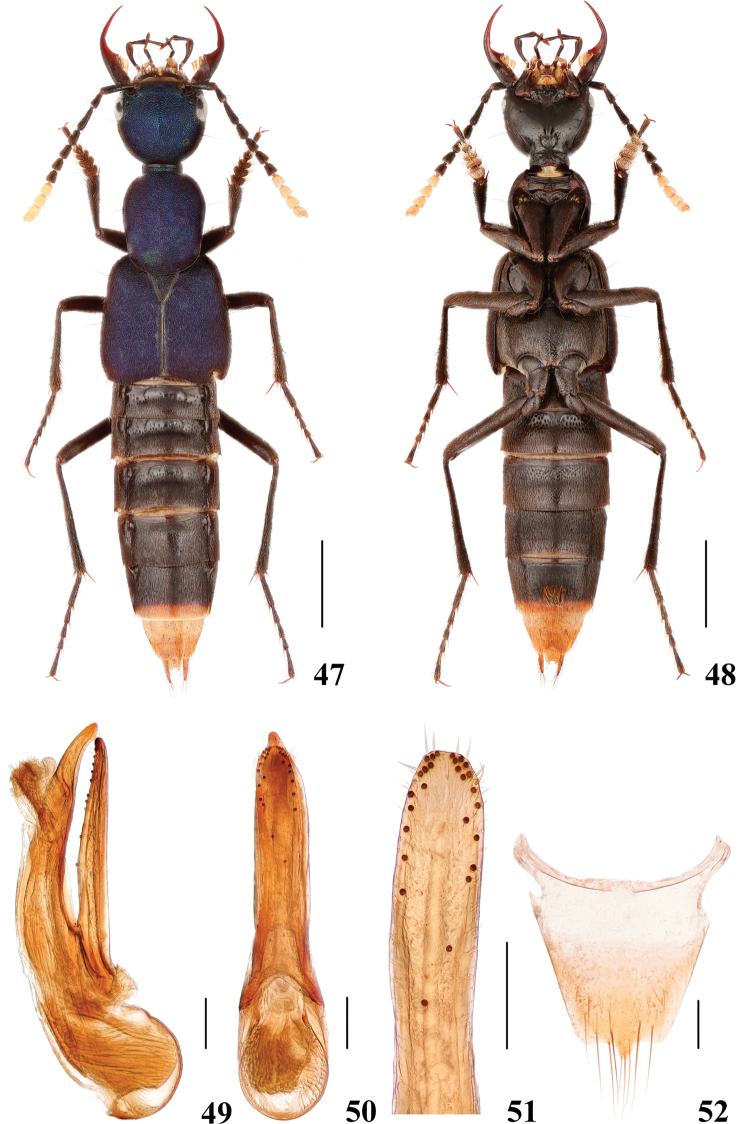
*Hesperosomapuetzi***47–48** habitus **49–51** aedeagus, lateral (**49**) and ventral (**50**) views, paramere (**51**) **52** female abdominal tergite X. Scale bars: 2 mm (**47–48**), 0.2 mm (**49–52**).

##### ﻿ Hesperosoma (s.str.) xizangense sp. nov.

Taxon classificationAnimaliaColeopteraStaphylinidae

789A0AEE-937F-5B98-BFAB-C0568AEAD456

http://zoobank.org/30F6B5FA-7004-4F4C-BA23-F0DB3E3A1971

Figures 11 , 53–57

###### Material examined.

***Holotype*.** China – **Xizang Prov.** • ♂; glued on a card with labels as follows: “China: Xizang, Bomi County, Yigong; alt. 2397 m; 27 Jul 2016; Yan-Quan Lu leg.; mixed forest.” “Holotype / Hesperosoma(s.str.)xizangense / Cai, Tang & Schillhammer” [red handwritten label]; SHNU.

###### Description.

**Measurements of male**: BL: 13.67 mm, FL: 8.12 mm. HL: 2.26 mm, HW: 2.91 mm, EYL: 0.74 mm, TL: 1.24 mm, PL: 2.75 mm, PW: 2.34 mm, EL: 3.55 mm, EW: 3.59 mm. HW/HL: 1.29, TL/EYL: 1.68, PL/PW: 1.18, EL/EW: 0.99.

Head metallic dark blue to violaceous blue, pronotum and elytra metallic dark blue to violaceous with purplish hue; abdomen with segments III–VI black, segment VII black with posterior margin broadly reddish-yellow, segments VIII and X entirely reddish-yellow, segment IX reddish-yellow with apical half of latter blackish; antennae black, base and apex of segment 1 and 2 reddish, four outer segments creamy white; mandibles black, medial margin and distal portion of mandible dark reddish-brown; maxillary and labial palpi deep black, last segments sometimes slightly paler brownish.

Head (Fig. 11) 1.29 times as wide as long, rounded trapezoid, tempora narrowed towards neck in regular arc, eyes moderately protruding; surface with dense and coarse punctation, mostly contiguous; with short, weakly delimited impunctate mid-line, extending from frons to about half of mid-length; antennae with segments 4–8 markedly oblong, segments 9 and 10 about as long as wide.

Pronotum 1.18 times as long as wide, widest at level of large lateral seta, narrowed towards base in wide, but shallow concave arc; surface as densely and coarsely punctate as on head, with indistinct, short impunctate mid-line in posterior third; scutellum with dense and pit-like punctation, interstices forming small transverse rugae.

Elytra as long as wide, exceedingly densely, coarsely punctate, punctures almost contiguous.

Abdominal tergites III–V with basal transverse depression, punctation of abdominal tergites III–V large, but feeble at base; posterior halves of abdominal tergites III–V and entire surface of remaining tergites with very fine and dense punctuation.

**Male.** Protarsomeres 1–4 moderately dilated, heart-shaped; sternite VII with patch of long yellow setae on median portion and posterior margin broadly emarginate at middle; sternite VIII with posterior margin emarginate at middle; aedeagus (Figs 55–57) very similar to that of *H.puetzi* and *H.xizangense* sp. nov., but markedly larger, median lobe and paramere slightly asymmetrical, paramere (Fig. 57) shorter than median lobe and slightly bent to left side in ventral view.

**Figures 53–57.**
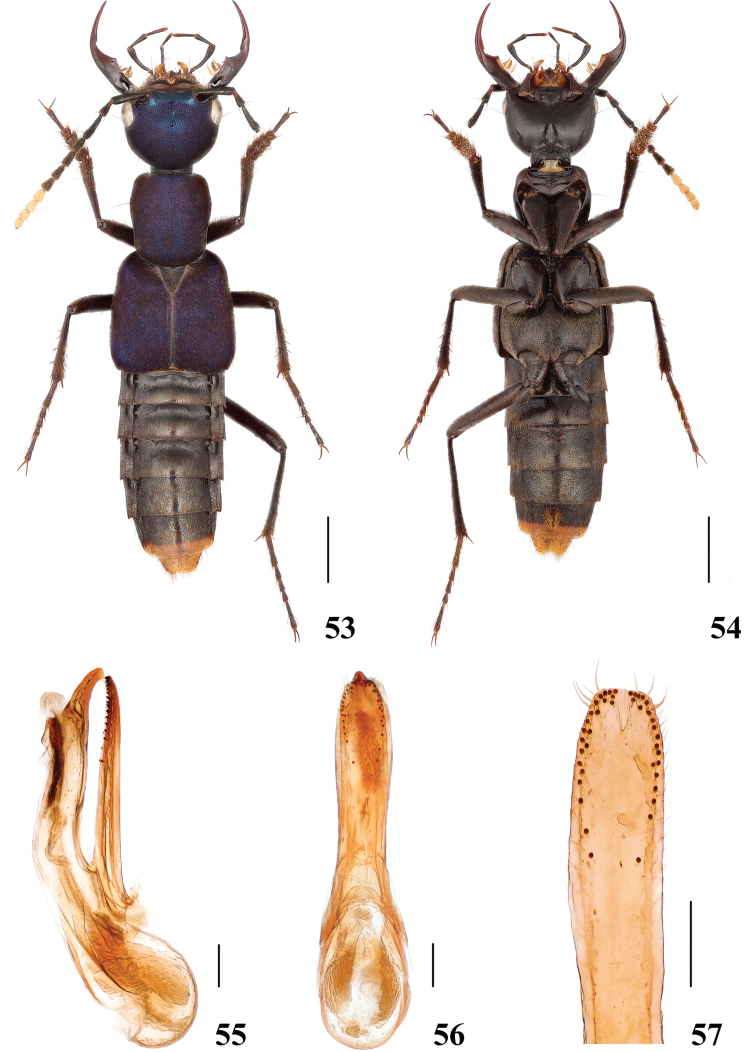
*Hesperosomaxizangense* sp. nov. **53–54** habitus **55–57** aedeagus, lateral (**55**) and ventral (**56**) views, paramere (**57**). Scale bars: 2 mm (**53–54**), 0.2 mm (**55–57**).

**Female.** Unknown.

###### Etymology.

The species is named after the type locality

###### Distribution.

China (Xizang).

###### Diagnosis.

The new species is very similar externally to *H.languidum* sp. nov. (Yunnan) and *H.puetzi* (Sichuan), but can be recognised by the emarginated anterior margin of frons (Figs 8–9), as well as by the different shape of the aedeagus, especially the paramere, which is broad and truncate apically. Additionally, the head is wider, 1.29 times as wide as long (1.14–1.16 in *H.puetzi*, 1.22 in *H.languidum* sp. nov.), the mandibles are relatively darker (relatively lighter in *H.puetzi* and *H.languidum* sp. nov.) and the legs are relatively darker (relatively lighter in *H.puetzi* and *H.languidum* sp. nov.).

#### Subgenus Paramichrotus

##### ﻿ Hesperosoma (Paramichrotus) alexpuchneri

Taxon classificationAnimaliaColeopteraStaphylinidae

Schillhammer, 2009

5C51329A-6F35-5775-9281-05B219D3CA2C

Figures 58–60

Hesperosoma alexpuchneri Schillhammer 2009: 87; Schillhammer 2014: 208
Hesperosoma (Hemihesperosoma) alexpuchneri Schillhammer 2015: 127

###### Material examined.

China – **Sichuan Prov.** • 1♀; Tianquan County, Lianglu Countryside; alt. 1400 m; 1 Aug 2011; Hao Huang leg.; SHNU.

###### Measurements.

Female. BL: 10.89 mm, FL: 5.28 mm. HL: 1.48 mm, HW: 1.89 mm, EYL: 0.56 mm, TL: 0.71 mm, PL: 1.76 mm, PW: 1.61 mm, EL: 2.29 mm, EW: 2.54 mm. HW/HL: 1.28, TL/EYL: 1.27, PL/PW: 1.09, EL/EW: 0.90.

###### Female characters.

Tergite X (Fig. 60) indistinctly asymmetrical with short apex.

###### Distribution.

China (Sichuan).

###### Diagnosis.

The collecting locality of the female specimen is about 68 km away from the type locality. The specimen corresponds with the original description and is, thus, temporarily assigned to *H.alexpuchneri*.

**Figures 58–60.**
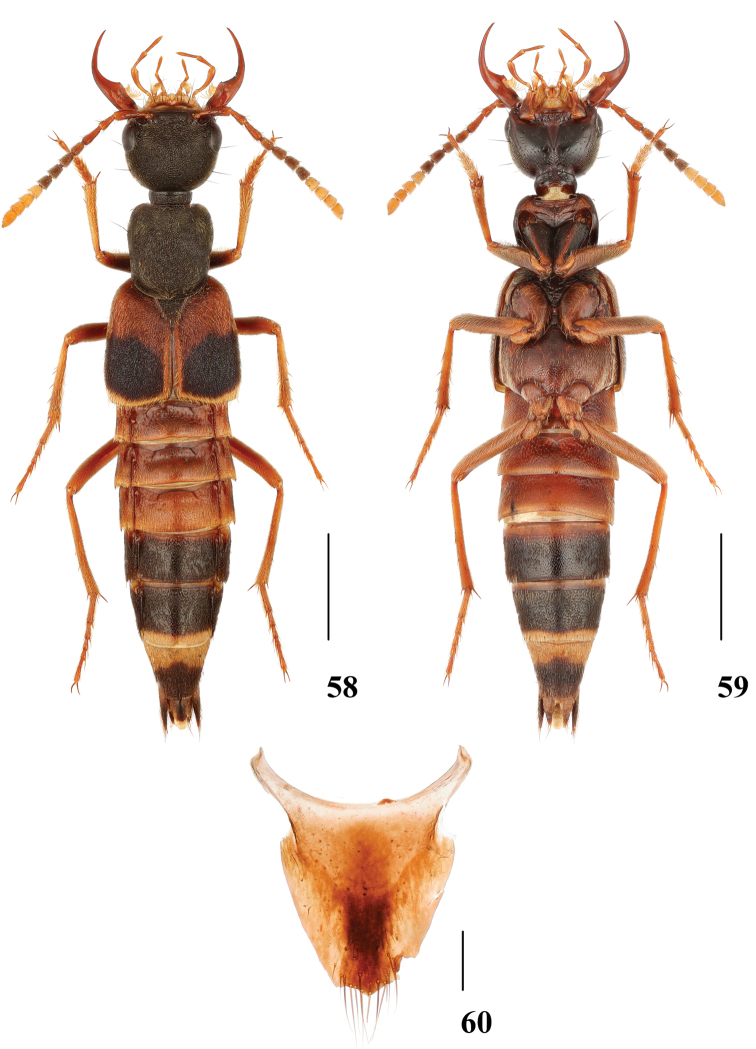
*Hesperosomaalexpuchneri***58–59** habitus **60** female abdominal tergite X. Scale bars: 2 mm (**58–59**), 0.2 mm (**60**).

##### ﻿ Hesperosoma (Paramichrotus) brunkei

Taxon classificationAnimaliaColeopteraStaphylinidae

Schillhammer, 2015

368D6787-3257-59DC-A0E6-42275E1C73ED

Figures 61–66

Hesperosoma (Hemihesperosoma) brunkei Schillhammer 2015: 133

###### Material examined.

China – **Yunnan Prov.** • 1♂; Dehon District, Ruili City, Ruili Botanical Garden; alt. 1100 m; 03 May 2013; Wen-Xuan Bi leg.; SHNU • 1♀; Dehongmang, Yingjiang County, Tongbiguan Town, near Mangna Road; 24°36'59"N, 97°44'30"E; alt. 1550 m; 29 Jul 2019; Cheng & Shen leg.; mixed leaf litter; sifted; SHNU • 1♀; Dehongmang, Yingjiang County, Xima Town, near Yingxi Road; 24°37'53"N, 97°45'39"E; alt. 1469 m; 1 Aug 2019; Cheng & Shen leg.; mixed leaf litter; sifted; SHNU • 1♀; Nabanhe N. R., Chuguohe, Bengganghani; alt. 1750 m; 28 Apr 2009; Jia-Yao Hu & Zi-Wei Yin leg.; SHNU.

###### Measurements.

**Male.**BL: 9.77 mm, FL: 5.59 mm. HL: 1.51 mm, HW: 1.92 mm, EYL: 0.64 mm, TL: 0.64 mm, PL: 2.04 mm, PW: 1.81 mm, EL: 2.45 mm, EW: 2.45 mm. HW/HL: 1.27, TL/EYL: 1.00, PL/PW: 1.13, EL/EW: 1.00.

**Female.**BL: 8.95–10.58 mm, FL: 5.85–6.19 mm. HL: 1.51–1.58 mm, HW: 1.89–2.04 mm, EYL: 0.68–0.71 mm, TL: 0.60–0.68 mm, PL: 2.07–2.19 mm, PW: 1.77–1.89 mm, EL: 2.57–2.72 mm, EW: 2.68–2.87 mm. HW/HL: 1.25–1.30, TL/EYL: 0.85–1.00, PL/PW: 1.14–1.17, EL/EW: 0.92–0.96.

###### Distribution.

China (Yunnan), Laos and Myanmar. New to China.

###### Diagnosis.

The collecting locality of the male specimen is about 760 km away from the holotype locality in north-eastern Laos and about 210 km away from the paratype locality in Myanmar. The specimen fits the original description in all characters, but the medio-apical margin of the paramere looks slightly different as well as the apex of the median lobe in lateral view. The ventral view of the median lobe as depicted here is more representative of the species, as it is matching that of the other specimens of the type series. The shape of the paramere is here considered as within the variability range of this widespread species. The females correspond with the original description in all characters.

**Figures 61–66.**
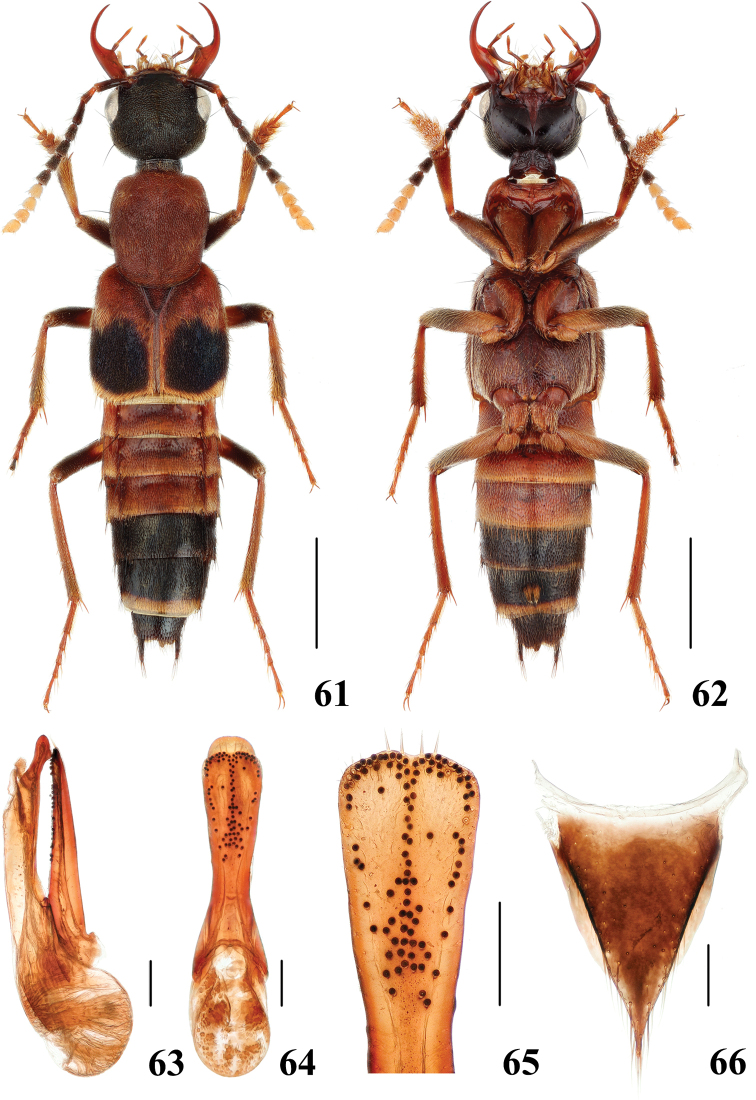
*Hesperosomabrunkei***61–62** habitus **63–65** aedeagus, lateral (**63**) and ventral (**64**) views, paramere (**65**) **66** female abdominal tergite X. Scale bars: 2 mm (**61–62**), 0.2 mm (**63–66**).

##### ﻿ Hesperosoma (Paramichrotus) excellens

Taxon classificationAnimaliaColeopteraStaphylinidae

(Bernhauer, 1939)

FC5B4B7D-122F-57E5-A004-2B182666D3C6

Figures 67–72

Amichrotus excellens Bernhauer 1939: 100
Hesperosoma (Euhesperosoma) excellens Hayashi 2002: 178; Schillhammer 2004: 261; Schillhammer 2009: 86
Hesperosoma (Hemihesperosoma) excellens Schillhammer, 2015: 134

###### Material examined.

China – **Anhui Prov.** • 1♂; Huangshan, Tangkou Town, Jiulongpu; 30°6'41.12"N, 118°12'37.74"E; alt. 760 m; 01 Jul 2020; Tang, Li & Zhou leg.; sifted; SHNU • 1♀; Huangshan, Tangkou Town, Hougu; 30°5'3.48"N, 118°8'45.96"E; alt. 569–688 m; 29 Jun –7 Jul 2020; Tang, Li & Zhou leg.; sifted; SHNU. – **Fujian Prov.** • 1♂; Wuyishan City, Guadun Vill; 27°44'N, 117°38'E; alt. 1200–1300 m; 24 May 2012; Peng & Dai leg.; SHNU • 1♂, 1♀; same collection data as for the preceding; but alt. 1200–1500 m; 25 May 2012 • 2♂♂; same collection data as for the preceding; but alt. 1200–1500 m; 26 May 2012 • 1♂; same collection data as for the preceding; but alt. 1300–1500 m; 27 May 2012. – **Guangdong Prov.** • 3♂♂; Ruyuan County, Nanling N. R.,Qingshui Valley; 24°54'57"N, 113°01'55"E; alt. 900 m; 04 May 2015; Peng, Tu & Zhou leg.; mixed forest, leaf litter, sifted; SHNU • 1♀; Ruyuan County, Nanling N. R.,Babaoshan station; 24°55'43"N, 113°00'58"E; alt. 1030 m; 25 Apr 2015; Z Peng, YY Tu & ZD Zhou leg.; decaying log; SHNU. – **Guangxi Prov.** • 1 ♂; Jinxiu County, ’16 km’; 24°08'11"N, 110°14'28"E; alt. 960 m; 25 Jul 2014; Peng, Song, Yu & Yan leg.; forest, leaf litter, sifted; SHNU • 1♂, 1♀; Jinxiu County, ’16 km’; 24°08'25"N, 110°15'38"E; alt. 960 m; 13 Jul 2014; Peng, Song, Yu & Yan leg.; beech forest, leaf litter, humus, sifted; SHNU. – **Hunan Prov.** • 1♂, 1♀; Yizhang County, Mang Mt.; 24°55'39"N, 112°59'28"E; alt. 1000–1200 m; 10 May 2020; Li & Wang leg.; FIT; SHNU. – **Zhejiang Prov.** • 1♂; Wuyanling; alt. 700 m; 9 May 2004; Hu, Tang & Zhu leg.; SHNU • 2♂♂; Longquan, Fengyang Mt., Guan Yin Tai; 27°55'23"N, 119°11'26"E; alt. 1100 m; 11 May 2019; Tang & Zhao leg.; sifted; SHNU • 1♂; Longquan, Fengyang Mt., Mihou Valley; 27°55'2"N, 119°11'37"E; alt. 950 m; 09 May 2019; Tang & Zhao leg.; sifted; SHNU • 1♂; same collection data as for the preceding; but 11 May 2019 • 1♀; Longquan City, Fengyangshan N. R., Lu’aocun Village; 27°55'19.66"N, 119°11'38.86"E; alt. 1076 m; 04 May 2016; Jiang, Liu & Zhou leg.; SHNU.

###### Measurements.

**Male.**BL: 10.23–13.98 mm, FL: 5.85–7.10 mm. HL: 1.61–1.89 mm, HW: 2.01–2.57 mm, EYL: 0.68–0.77 mm, TL: 0.68–0.86 mm, PL: 2.17–2.60 mm, PW: 1.76–2.23 mm, EL: 2.51–3.17 mm, EW: 2.57–3.32 mm. HW/HL: 1.25–1.36, TL/EYL: 1.00–1.16, PL/PW: 1.14–1.23, EL/EW: 0.95–0.99.

**Female.**BL: 11.72–13.36 mm, FL: 6.48–7.22 mm. HL: 1.71–1.92 mm, HW: 2.23–2.41 mm, EYL: 0.71–0.77 mm, TL: 0.77–0.83 mm, PL: 2.32–2.60 mm, PW: 2.01–2.23 mm, EL: 2.85–3.32 mm, EW: 2.91–3.32 mm. HW/HL: 1.25–1.30, TL/EYL: 1.00–1.13, PL/PW: 1.14–1.17, EL/EW: 0.96–1.00.

###### Distribution.

China (Anhui, Fujian, Guangdong, Guangxi, Hubei and Zhejiang). New to Anhui, Guangdong, Guangxi and Zhejiang.

###### Diagnosis.

**Figures 67–72.**
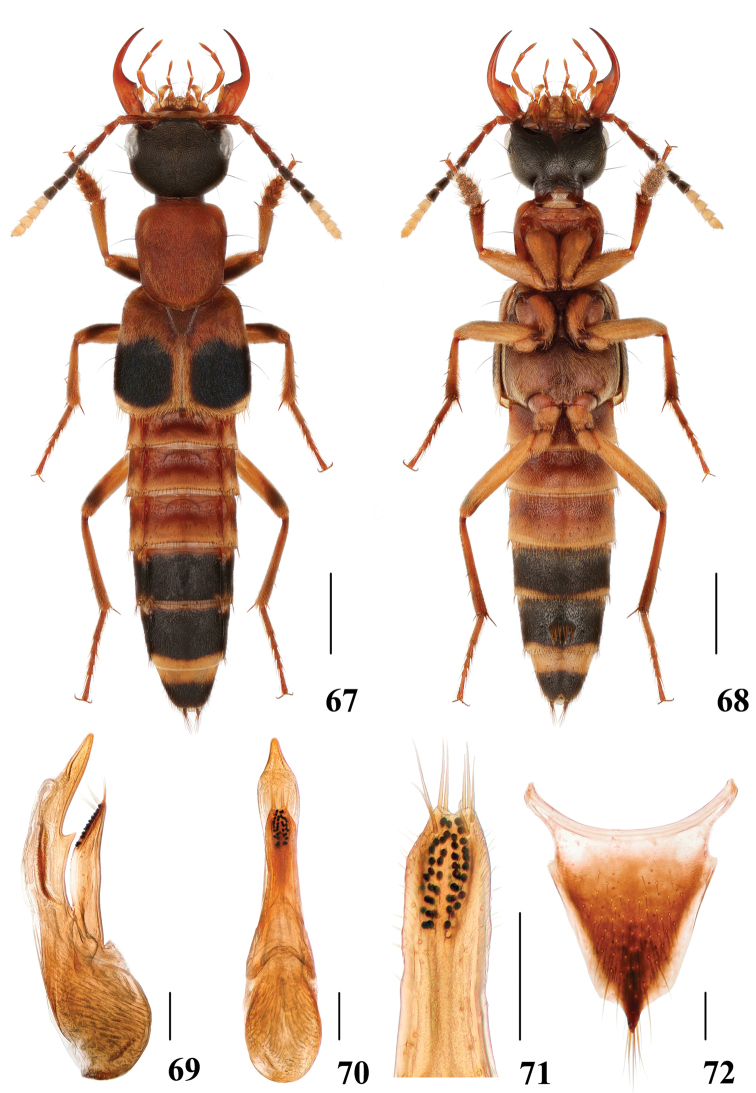
*Hesperosomaexcellens***67–68** habitus **69–71** aedeagus, lateral (**69**) and ventral (**70**) views, paramere (**71**) **72** female abdominal tergite X. Scale bars: 2 mm (**67–68**), 0.2 mm (**69–72**).

Externally, the species hardly differs from *H.meghalayense* from India, but may be distinguished by the very different aedeagus (Figs 67–69) and, to some extent, geographically. From *H.brunkei*, it differs in the proximal antennomeres 3 and 4 being bright reddish and segments I–IV of maxillary palpi reddish.

##### ﻿ Hesperosoma (Paramichrotus) guizhouense

Taxon classificationAnimaliaColeopteraStaphylinidae

Schillhammer, 2018

D293ABCA-9FD1-504F-86EC-C109C025C46A

Figures 73–78

Hesperosoma guizhouense Schillhammer 2018: 44

###### Material examined.

China – **Guizhou Prov.** • 1♂; Rongjiang County, Xiaodanjiang; 26°20'16.09"N, 108°20'23.34"E; alt. 700 m; 5 May 2021; De-Yao Zhou leg.; SHNU • 1♀; Leishan County, Leigong Mt., Xiannütang; 26°22'22.11"N, 108°11'52.12"E; alt. 1550 m; 30 Apr 2021; Tang, Peng, Cai & Song leg.; SHNU.

###### Measurements.

**Male.**BL: 11.19 mm, FL: 6.17 mm. HL: 1.67 mm, HW: 2.29 mm, EYL: 0.71 mm, TL: 0.74 mm, PL: 2.17 mm, PW: 1.86 mm, EL: 2.76 mm, EW: 2.66 mm. HW/HL: 1.37, TL/EYL: 1.04, PL/PW: 1.17, EL/EW: 1.04.

**Female.**BL: 11.47 mm, FL: 6.11 mm. HL: 1.67 mm, HW: 2.17 mm, EYL: 0.65 mm, TL: 0.83 mm, PL: 2.11 mm, PW: 1.86 mm, EL: 2.69 mm, EW: 2.76 mm. HW/HL: 1.30, TL/EYL: 1.28, PL/PW: 1.13, EL/EW: 0.97.

###### Female characters.

Tergite X (Fig. 78) slightly asymmetrical with blunt apex.

###### Distribution.

China (Guizhou).

###### Diagnosis.

**Figures 73–78.**
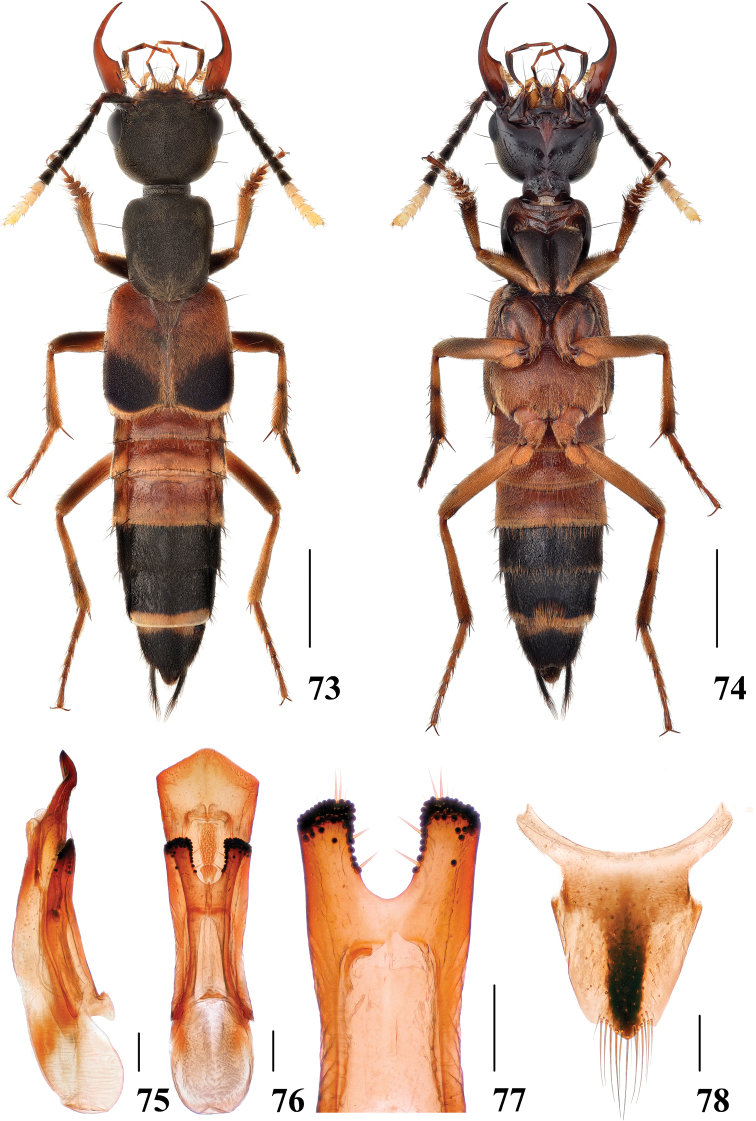
*Hesperosomaguizhouense***73–74** habitus **75–77** aedeagus, lateral (**75**) and ventral (**76**) views, paramere (**77**) **78** female abdominal tergite X. Scale bars: 2 mm (**73–74**), 0.2 mm (**75–78**).

*Hesperosomaguizhouense* does not differ externally from two allopatric species, *H.klapperichi* and *H.alexpuchneri*, but it may be distinguished only by the shape of the aedeagus (Figs 73–75): paramere in *H.guizhouense* with relatively deeper medio-apical emargination, in *H.klapperichi* with relatively shallower medio-apical emargination and in *H.alexpuchneri* with acutely pointed apex. In one examined male specimen, the suture is reddish, which may be explained by the variability of the species.

##### ﻿ Hesperosoma (Paramichrotus) klapperichi

Taxon classificationAnimaliaColeopteraStaphylinidae

Schillhammer, 2004

8A9A1F0B-C6E9-599E-B0FB-0929F6B70AE7

Figures 79–84

Hesperosoma klapperichi Schillhammer 2004: 260
Hesperosoma (Hemihesperosoma) klapperichi Schillhammer, 2015: 126

###### Material examined.

China – **Anhui Prov.** • 2♂♂, 1♀; Huangshan, Tangkou Town, Hougu; 30°5'3.48"N, 118°8'45.96"E; alt. 569–688 m; 29 Jun –3 Jul 2020; Chong Li leg.; pitfall trapped; SHNU. – **Fujian Prov.** • 1♂; Wuyishan City, Guadun Vill; 27°44'N, 117°38'E; alt. 1200–1500 m; 26 May 2012; Peng & Dai leg.; SHNU. – **Guangxi Prov.** • 7♂♂, 1♀; Jinxiu County, ’16 km’; 24°08'25"N, 110°15'38"E; alt. 960 m; 13 Jul 2014; Peng, Song, Yu & Yan leg.; beech forest, mixed leaf litter, humus, shifted; SHNU • 1♀; Jinxiu County, ’16 km’; 24°08'11"N, 110°14'28"E; alt. 960 m; 25 Jul 2014; Peng, Song, Yu & Yan leg.; forest, leaf litter, shifted; SHNU • 1♂, 1♀; Jinxiu County, Laoshan Forest Farm; 24°07'17"N, 110°11'54"E; alt. 840 m; 18 Jul 2014; Peng, Song, Yu & Yan leg.; beech forest, mixed leaf litter, humus, shifted; SHNU • 1♀; Lingui County, Huaping, Anjiangping; alt. 1200 m; 16 Jul 2011; L Tang & W-J He leg.; SHNU • 2♂♂; Jinxiu County, Dayaoshan, Luoyingou; alt. 1200 m; 15 Jul 2016; Jin-Teng Zhao leg.; SHNU • 1♀; Jinxiu County, Yinshan Conservation Station; 24°10'01"N, 110°14'38"E; alt. 1200 m; 10 Jul 2014; Peng, Song, Yu & Yan leg.; beech forest, mixed leaf litter, shifted; SHNU. – **Hunan Prov.** • 1♀; Xinning County, Shunhuang Mt., Yangheping; 26°23'41.58"N, 111°00'08.16"E; alt. 820 m; 2 May 2021; Yin, Zhang, Pan & Shen leg.; SHNU.

###### Measurements.

**Male.**BL: 9.51–12.15 mm, FL: 5.82–6.69 mm. HL: 1.55–1.82 mm, HW: 2.20–2.79 mm, EYL: 0.65–0.74 mm, TL: 0.71–0.93 mm, PL: 2.07–2.32 mm, PW: 1.79–2.13 mm, EL: 2.57–3.07 mm, EW: 2.51–3.01 mm. HW/HL: 1.39–1.53, TL/EYL: 1.04–1.31, PL/PW: 1.09–1.17, EL/EW: 1.00–1.02.

**Female.**BL: 10.14–12.77 mm, FL: 6.07–6.66 mm. HL: 1.61–1.79 mm, HW: 2.13–2.38 mm, EYL: 0.65–0.74 mm, TL: 0.74–0.86 mm, PL: 2.13–2.38 mm, PW: 1.89–2.07 mm, EL: 2.54–2.97 mm, EW: 2.69–3.10 mm. HW/HL: 1.30–1.44, TL/EYL: 1.08–1.17, PL/PW: 1.13–1.16, EL/EW: 0.94–0.98.

###### Distribution.

China (Anhui, Fujian, Guangxi, Hubei and Hunan). New to Anhui, Guangxi and Hunan.

###### Diagnosis.

The species is very similar to *H.miwai*, but may be distinguished by the paler, almost entirely reddish tibiae. From *H.guizhouense* and *H.alexpuchneri*, the main distinguishing character is the aedeagus (Figs 81–83) (see diagnosis in *H.guizhouense*).

**Figures 79–84.**
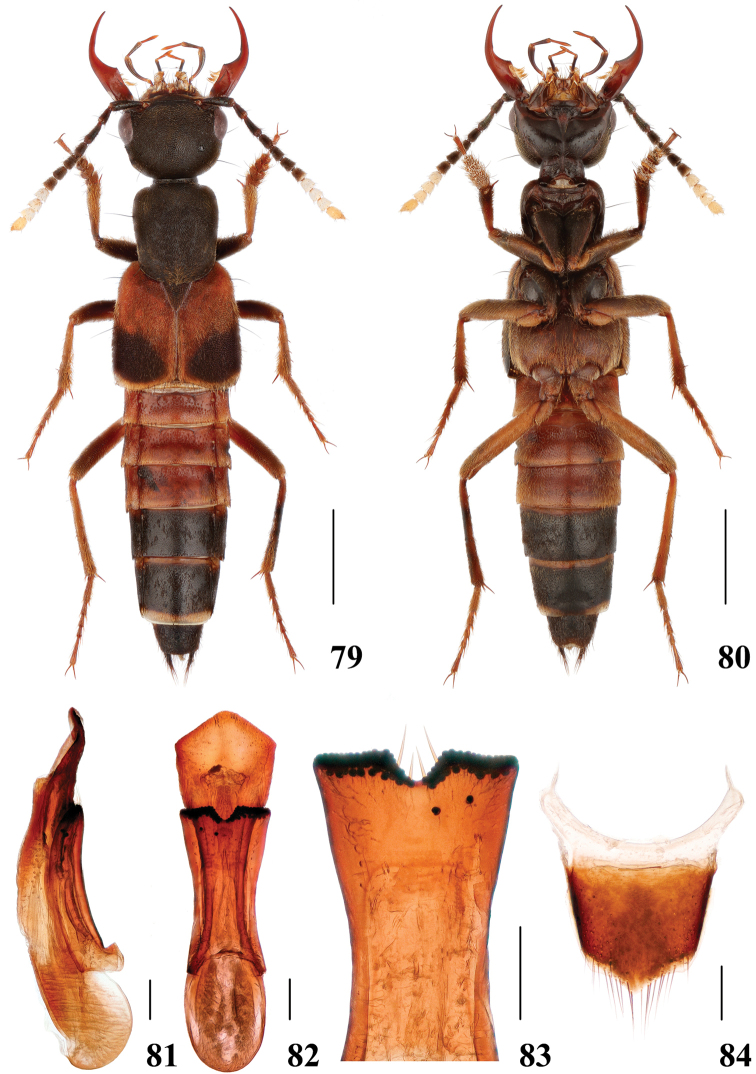
*Hesperosomaklapperichi***79–80** habitus **81–83** aedeagus, lateral (**81**) and ventral (**82**) views, paramere (**83**) **84** female abdominal tergite X. Scale bars: 2 mm (**79–80**), 0.2 mm (**81–84**).

##### ﻿ Hesperosoma (Paramichrotus) miwai

Taxon classificationAnimaliaColeopteraStaphylinidae

(Bernhauer, 1943)

41F730D1-BC0D-53BC-AF3B-99FC99640E57

Figures 85–89

Amichrotus miwai Bernhauer 1943: 177; Shibata 1976: 11
Hesperosoma miwai Hayashi 1993a: 290; Hayashi 2002: 177; Schillhammer 2009: 86
Hesperosoma (Hemihesperosoma) miwai Schillhammer, 2015: 125
Hesperosoma miwai nashanchiana Hayashi, 1993b: 123; Schillhammer 2009: 86
Hesperosoma sakoi Hayashi, 1993b: 124; Schillhammer 2009: 86

###### Material examined.

China – **Taiwan** • 1♂; Pingtung, Tai-wu, Pei-ta-wu-shan; 22°37'47"N, 120°45'32"E; alt. 1300 m; 10 Oct 2017; Chung leg.; SHNU.

###### Measurements.

Male. BL: 10.73 mm, FL: 5.82 mm. HL: 1.62 mm, HW: 2.15 mm, EYL: 0.64 mm, TL: 0.79 mm, PL: 1.96 mm, PW: 1.77 mm, EL: 2.49 mm, EW: 2.41 mm. HW/HL: 1.33, TL/EYL: 1.23, PL/PW: 1.11, EL/EW: 1.03.

###### Distribution.

China (Taiwan).

###### Diagnosis.

Externally, the species is very similar to *H.guizhouense*, *H.klapperichi* and *H.alexpuchneri*, both in colouration and shape, but it differs mainly, in addition to the aedeagus (Figs 87–89) and geography, by all tibiae being predominantly black.

**Figures 85–89.**
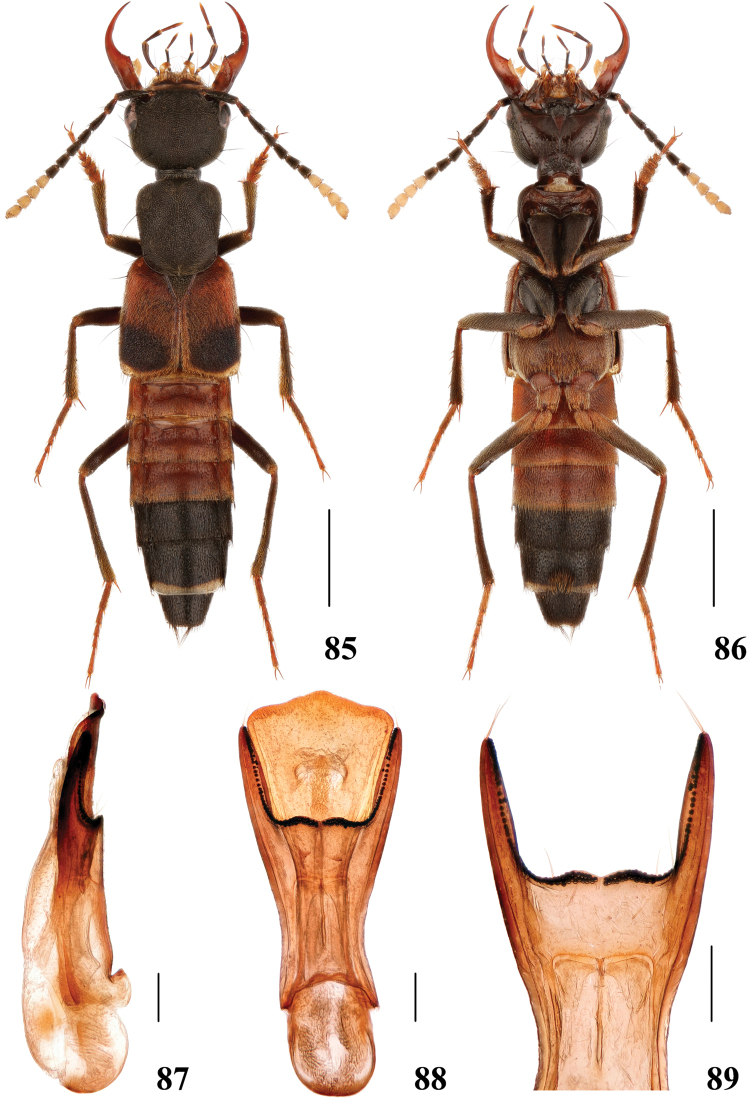
*Hesperosomamiwai***85–86** habitus **87–89** aedeagus, lateral (**87**) and ventral (**88**) views, paramere (**89**). Scale bars: 2 mm (**85–86**), 0.2 mm (**87–89**).

##### ﻿ Hesperosoma (Paramichrotus) parvioculatum sp. nov.

Taxon classificationAnimaliaColeopteraStaphylinidae

A3E046B4-4071-5ADA-A433-A2B432BD1590

http://zoobank.org/616E3B86-58DA-4D44-BEBD-C30DE0FEB289

Figures 90–95

###### Material examined.

***Holotype*.** China – **Hunan Prov.** • ♂; glued on a card with labels as follows: “China: Hunan, Yanling County, Nanfengmian; 26°18'10"N, 114°00'12"E; alt. 1620 m; 26 May 2014; Peng, Shen, Yu & Yan leg.; mixed forest, leaf litter, wood sifted” “Holotype / Hesperosoma (Paramichrotus) parvioculatum / Cai, Tang & Schillhammer” [red handwritten label]; SHNU. ***Paratypes*.** China – **Hunan Prov.** • 1♂; Yizhang County, Mang Mt.; 24°55'39"N, 112°59'28"E; alt. 1000–1200 m; 10 May 2020; Li & Wang leg.; FIT; NMW • 1♀; same collection data as for the preceding; but 17 Jul 2020; SHNU. – **Hubei Prov.** • 1♂, Wufeng County, Houhe Natural Reserve; 30°05'09"N, 110°33'05"E; alt. 1160 m; 08 Jul 2013; Dai, Peng & Xie leg.; along path in a mixed forest; bamboo; leaf litter; sifted; SHNU.

###### Description.

**Measurements of male**: BL: 10.61–13.33 mm, FL: 6.35–6.73 mm. HL: 1.58–1.79 mm, HW: 2.57–2.79 mm, EYL: 0.62–0.71 mm, TL: 0.86–0.96 mm, PL: 2.15–2.38 mm, PW: 1.95–2.11 mm, EL: 2.85–3.04 mm, EW: 2.83–3.10 mm. HW/HL: 1.48–1.63, TL/EYL: 1.35–1.39, PL/PW: 1.08–1.13, EL/EW: 0.98–1.01.

**Measurements of female**: BL: 13.79 mm, FL: 6.82 mm. HL: 1.83 mm, HW: 2.384 mm, EYL: 0.71 mm, TL: 0.92 mm, PL: 2.42 mm, PW: 2.07 mm, EL: 3.01 mm, EW: 3.13 mm. HW/HL: 1.30, TL/EYL: 1.30, PL/PW: 1.17, EL/EW: 0.96.

Head, pronotum and scutellum black; elytra red, with a large black patch with faint bluish hue, nearly occupying posterior two thirds, continuing on to hypomeron, but not reaching ventro-lateral margin, posterior margin and suture narrowly yellowish-red; abdomen with segments III–V reddish, VI black with anterior margin narrowly reddish, VII black with posterior margin narrowly yellowish, VIII with proximal half yellowish and distal half black, IX dark brown, X black, narrowly, obscurely reddish at base; antennae with segments 1–7 black, base and apex of segment 1, 2 and base of segment 3 reddish, segments 8–11 creamy white; mandibles dark reddish-brown; maxillary palpi reddish, labial palpi with segments 1 and 3 reddish, segment 2 black brown; legs reddish-yellow, femora reddish-brown.

Head 1.48–1.63 times as long as wide, rounded trapezoid, tempora narrowed behind eyes; dorsal surface coarsely and very densely punctate, punctures contiguous, including clypeus; antennae rather short, antennae with segments 4–7 weakly oblong, segments 8 and 10 about as long as wide.

Pronotum 1.08–1.13 times as long as wide, widest at about level of large antero-lateral seta, narrowed towards base in weak concave arc, surface with dense and coarse punctation similar to that on head, with narrow impunctate mid-line, ground pubescence slightly more obvious than that on head; scutellum uniformly, densely punctate, space between punctures with very fine wavy microsculpture.

Elytra as long as wide, along sides distinctly longer than pronotum, with slightly uneven surface, densely punctate and pubescent, with distinct depression between shoulders, scutellum and apical margin of elytra; pubescence yellow, long and dense along suture and posterior elytral margin.

Abdominal tergites III–V with transverse basal depression and pair of short oblique basal carinae, punctation very sparse and rather coarse basally and laterally, gradually becoming finer towards apical margin, tergites VI–VIII entirely with fine and uniform punctation and pubescence, colour of pubescence corresponding with colour of integument underneath; legs long and slender.

**Male.** Protarsomeres 1–4 moderately dilated, heart-shaped; sternite VII with the usual setose groove and patch of long yellow setae on median portion, posterior margin broadly emarginate at middle; sternite VIII with posterior margin emarginate at middle; aedeagus (Figs 92–94) with apex of median lobe broad, anterior margin with gibbosities at middle (ventral view); paramere (Fig. 94) with narrow apical portion, shorter than median lobe.

**Female.** Tergite X (Fig. 95) slightly asymmetrical with short apex.

**Figures 90–95.**
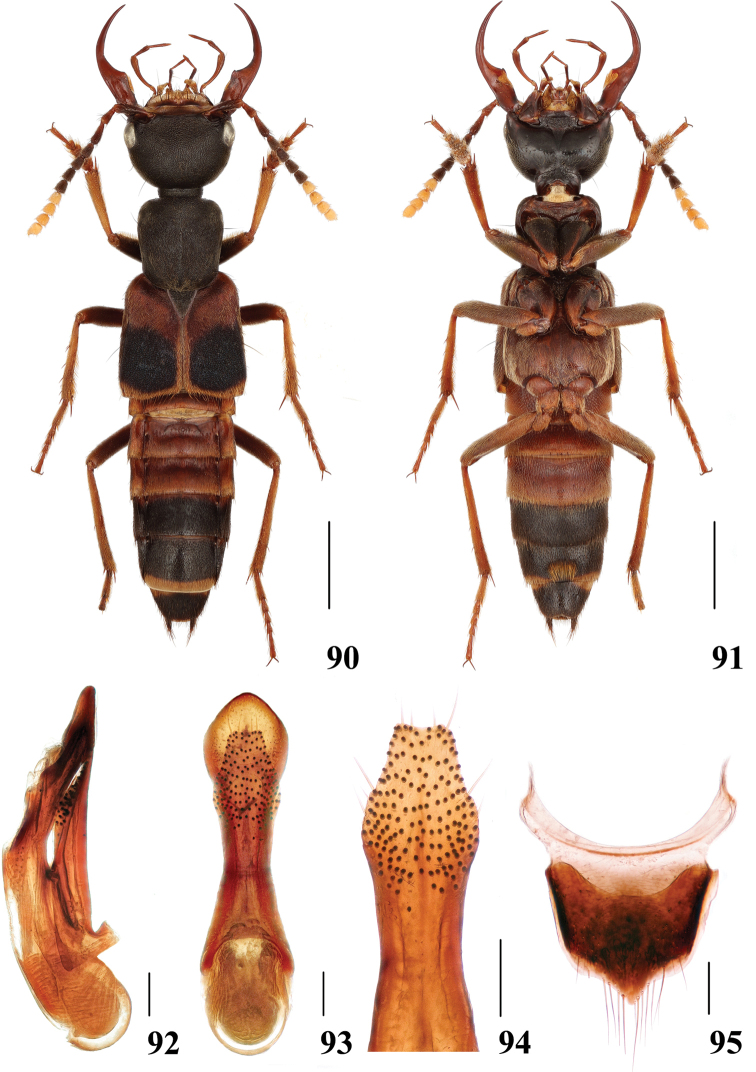
*Hesperosomaparvioculatum* sp. nov. **90–91** habitus **92–94** aedeagus, lateral (**92**) and ventral (**93**) views, paramere (**94**) **95** female abdominal tergite X. Scale bars: 2 mm (**90–91**), 0.2 mm (**92–95**).

###### Etymology.

The specific epithet refers to the small eyes of the new species.

###### Distribution.

China (Hubei and Hunan).

###### Diagnosis.

Externally, *H.parvioculatum* sp. nov. is virtually identical to *H.nigricolle* from Myanmar, but differs mainly, aside from the distribution, by the shape of the aedeagus: paramere with narrow apical portion (paramere with broad apical portion, distinctly bilobed in *H.nigricolle*); the wider head with HW/HL about 1.48–1.63 (1.35 in *H.nigricolle*); posterior angles of the head being rounded (slightly prominent in *H.nigricolle*); legs reddish-yellow, femora reddish-brown (legs reddish to yellowish-red, distal halves of femora black in *H.nigricolle*). In appearance, it is also similar to *H.klapperichi*, *H.alexpuchneri*, *H.guizhouense* and *H.yunnanense*, but can be distinguished from *H.klapperichi*, *H.alexpuchneri* and *H.guizhouense* by the larger black elytral patch, occupying more than half of the elytral disc. From *H.yunnanense*, it is distinguished by the smaller eyes with TL/EYL about 1.35–1.39 (1.18–1.29 in *H.yunnanense*) and the wider head with HW/HL about 1.48–1.63 (1.26–1.40 in *H.yunnanense*).

##### ﻿ Hesperosoma (Paramichrotus) yunnanense

Taxon classificationAnimaliaColeopteraStaphylinidae

Schillhammer, 2009

77CE8620-092D-55A1-8DB0-4377E5CB60D3

Figures 96–101

Hesperosoma yunnanense Schillhammer 2009: 88; Schillhammer 2014: 208
Hesperosoma (Hemihesperosoma) yunnanense Schillhammer 2015: 128

###### Material examined.

China – **Yunnan Prov.** • 1♂, 1♀; Yinjiang County, Sudian Town, Maocaozhaicun; 25°08'10"N, 97°52'44"E; alt. 1900 m; 15–18 May 2020; Lu Qiu leg; SHNU • 1♂; Baoshan City, Baihualing; 25°16'46"N, 98°47'20"E; alt. 1350–1450 m; 22 Apr 2013; Song, Peng & Dai leg.; SHNU • 1♂; Nabanhe N. R., Bengganghani, Nanmugaha; alt. 1650 m; 30 Apr 2009; Jia-Yao Hu & Zi-Wei Yin leg.; SHNU • 1♂; Lingcang City., Wumulong, Xinfangzi; 25.17N, 99.69E; alt. 2450 m; 16 Jun 2015; Mao Ye leg.; SHNU • 1♀; Nabanhe N. R., Bengganghani; alt. 2000 m; 29 Apr 2009; Jia-Yao Hu & Zi-Wei Yin leg.; SHNU.

###### Measurements.

**Male.**BL: 9.76–10.73 mm, FL: 5.51–6.01 mm. HL: 1.45–1.67 mm, HW: 1.89–2.33 mm, EYL: 0.55–0.68 mm, TL: 0.71–0.89 mm, PL: 1.89–2.11 mm, PW: 1.70–1.92 mm, EL: 2.35–2.78 mm, EW: 2.38–2.79 mm. HW/HL: 1.30–1.40, TL/EYL: 1.18–1.38, PL/PW: 1.08–1.13, EL/EW: 0.96–1.00.

**Female.**BL: 10.72–11.56 mm, FL: 6.07–6.11 mm. HL: 1.58–1.64 mm, HW: 2.01–2.10 mm, EYL: 0.62–0.65 mm, TL: 0.77–0.80 mm, PL: 2.04–2.13 mm, PW: 1.88–1.89 mm, EL: 2.69–2.72 mm, EW: 2.79–2.82 mm. HW/HL: 1.27–1.28, TL/EYL: 1.18–1.29, PL/PW: 1.08–1.13, EL/EW: 0.95–0.96.

###### Distribution.

China (Yunnan).

###### Diagnosis.

Externally, the species is similar to *H.miwai*, *H.klapperichi*, *H.guizhouense* and *H.alexpuchneri*, both in colouration and shape and differs mainly in the much larger black elytral spot, occupying the apical two thirds of each elytron. For differences with *H.parvioculatum*, see diagnosis under that species.

**Figures 96–101.**
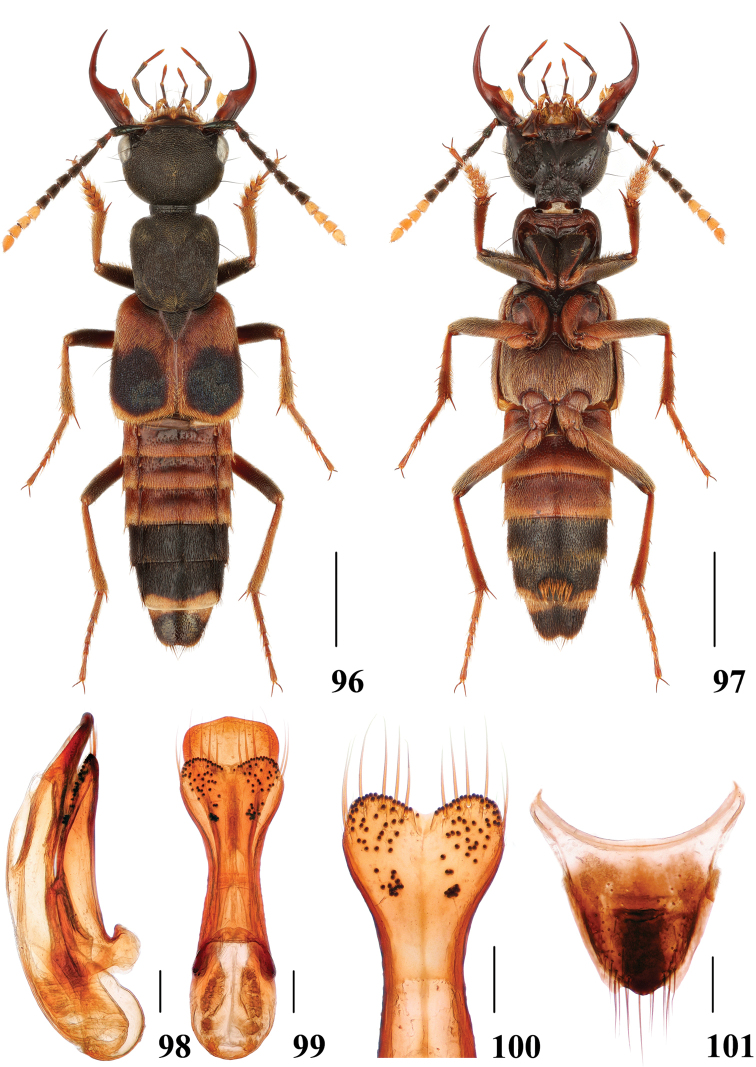
*Hesperosomayunnanense***96–97** habitus **98–100** aedeagus, lateral (**98**) and ventral (**99**) views, paramere (**100**) **101** female abdominal tergite X. Scale bars: 2 mm (**96–97**), 0.2 mm (**98–101**).

## Supplementary Material

XML Treatment for Hesperosoma (s.str.) chenchangchini

XML Treatment for Hesperosoma (s.str.) chinense

XML Treatment for Hesperosoma (s.str.) flavoterminale

XML Treatment for Hesperosoma (s.str.) kleebergi

XML Treatment for Hesperosoma (s.str.) languidum

XML Treatment for Hesperosoma (s.str.) motuoense

XML Treatment for Hesperosoma (s.str.) puetzi

XML Treatment for Hesperosoma (s.str.) xizangense

XML Treatment for Hesperosoma (Paramichrotus) alexpuchneri

XML Treatment for Hesperosoma (Paramichrotus) brunkei

XML Treatment for Hesperosoma (Paramichrotus) excellens

XML Treatment for Hesperosoma (Paramichrotus) guizhouense

XML Treatment for Hesperosoma (Paramichrotus) klapperichi

XML Treatment for Hesperosoma (Paramichrotus) miwai

XML Treatment for Hesperosoma (Paramichrotus) parvioculatum

XML Treatment for Hesperosoma (Paramichrotus) yunnanense
